# Portuguese Physical Literacy Assessment Questionnaire (PPLA-Q) for adolescents (15–18 years) from grades 10–12: development, content validation and pilot testing

**DOI:** 10.1186/s12889-021-12230-5

**Published:** 2021-11-29

**Authors:** João Mota, João Martins, Marcos Onofre

**Affiliations:** 1 Centro de Estudos de Educação, Faculdade de Motricidade Humana, Universidade de Lisboa, Estrada da Costa, Cruz-Quebrada-Dafundo, Oeiras, Portugal; 2 UIDEF, Instituto de Educação, Universidade de Lisboa, Alameda da Universidade, Lisbon, Portugal

**Keywords:** Physical literacy, Assessment, Physical education, Content validity, Pilot testing, High-school, Adolescence

## Abstract

**Background:**

The *Portuguese Physical Literacy Assessment* (PPLA) is a novel tool to assess high-school students’ (grade 10–12; 15–18 years) Physical Literacy (PL) in Physical Education (PE); inspired by the four domains of the *Australian Physical Literacy Framework* (APLF), and the Portuguese PE syllabus. This paper describes the development, content validation, and pilot testing of the PPLA-Questionnaire *(PPLA-Q)*, one of two instruments in the PPLA, comprised of modules to assess the *psychological*, *social,* and part of the *cognitive* domain of PL.

**Methods:**

Development was supported by previous work, analysis of the *APLF*, and literature review. We iteratively gathered evidence on content validity through two rounds of qualitative and quantitative expert validation (*n* = 11); three rounds of cognitive interviews with high-school students (*n* = 12); and multiple instances of expert advisor input. A pilot study in two grade 10 classes (*n* = 41) assessed feasibility, preliminary reliability, item difficulty and discrimination.

**Results:**

Initial versions of the PPLA-Q gathered evidence in favor of adequate content validity at item level: most items had an Item-Content Validity Index ≥.78 and Cohen’s **κ** ≥ .76. At module-level, S-CVI/Ave and UA were .87/.60, .98/.93 and .96/.84 for the cognitive, psychological, and social modules, respectively. Through the pilot study, we found evidence for feasibility, preliminary subscale and item reliability, difficulty, and discrimination. Items were reviewed through qualitative methods until saturation. Current PPLA-Q consists of 3 modules: cognitive (knowledge test with 10 items), psychological (46 Likert-type items) and social (43 Likert-type items).

**Conclusion:**

Results of this study provide evidence for content validity, feasibility within PE setting and preliminary reliability of the PPLA-Q as an instrument to assess the psychological, social, and part of the cognitive domain of PL in grade 10 to 12 adolescents. Further validation and development are needed to establish construct validity and reliability, and study PPLA-Q’s integration with the PPLA-Observation (an instrument in development to assess the remaining domains of PL) within the PPLA framework.

**Supplementary Information:**

The online version contains supplementary material available at 10.1186/s12889-021-12230-5.

## Background

Physical Literacy (PL) is a concept based on lifelong holistic learning acquired and applied in movement and physical activity (PA) contexts [1]. Arguably, the most seminal contribution to the development of the concept in modern pedagogy have been the works of Margaret Whitehead [2–4], which conceptualized PL as the motivation, confidence, physical competence, understanding and knowledge to maintain physical activity throughout the life course.

Notwithstanding its lifelong development, sowing the seeds of PL during school-age seems critical, as participation in early childhood might predict adherence to active lifestyles throughout life [5, 6], counteracting the rising levels of physical inactivity observed in adolescents and adults [7, 8]. In this line, PL is argued as the main outcome of quality physical education (PE) in schools [9], since it provides a privileged environment – mandatory, free and qualified – for learning the life skills and values needed for active and global citizenship [10]; as well as being the only opportunity to participate and learn from PA for some school-aged children and adolescents [11]. Thus, many authors have underlined the need to operationalize this concept in school curricula and educational policies [12–14].

Despite a general consensus on the ultimate goal of PL – sustained lifelong PA participation [15, 16] –, its proposed conceptualization and constituent elements differ across sources [17–19]. These range from philosophically-driven conceptualizations, like Whitehead’s PL original proposition [2] – rooted in the philosophical tenets of monism, phenomenology, and existentialism – to diametrical conceptualizations focusing solely on one of its aspects (e.g., fundamental movement skills) [20]. Although recognized as a rich theoretical concept, the former might lack pragmaticism to be implemented in practice [17]: while the later might deviate from the holistic nature of PL, compromising crucial elements like pleasure and enjoyment in taking part in PA [21]. As such, a middle-ground compromise might offer a tenable solution: providing clear and measurable outcomes, while honoring most of the philosophical-driven premises that define the concept [20, 22]. To this end, a team of Australia-based researchers developed the *Australian Physical Literacy Framework* (APLF) [1], a research-based, integrated model of PL in the *physical*, *cognitive*, *psychological* and *social* domains with 30 different elements – novel in recognizing the contribute that PL might play in cultural and social participation. It provides a clear focus on a learning continuum, inspired by the *Structure of Observed Learning Outcomes* taxonomy [23], designed to include individuals in different states of their PL journey: from their first steps (*pre-foundational*) to higher stages of proficiency (*transfer & empowerment*) [24, 25].

### Physical literacy assessment

Given evaluation’s essential role in PL implementation and practice [12] a few assessment instruments have been developed, under diverse conceptual models [18, 26, 27]. Of these, the most prolific research-wise have been the Canadian Assessment of Physical Literacy (CAPL) [28, 29], and the Physical Literacy Assessment for Youth [30] (PLAY). The CAPL is comprised of standardized assessments developed for children from 8 to 12 years [31] (with preliminary testing done in 12 to 16 year-olds [32]), to assess daily behavior, physical competence, motivation and confidence, and knowledge and understanding. The PLAY tools have been developed to assess children from 7 years up (with recommendations mainly targeted at the 7–12-year range), comprised of measures of motor competence, comprehension, and confidence. Both tools integrate observational procedures and self-report, and feature overall good feasibility in PE [27] but lack options for older adolescents (15–18 years), a critical age range in Portugal which presents lower levels of PA [33–35] – making them a priority target in the Portuguese PE setting.

### Portuguese physical education and PL

The Portuguese PE national syllabus (PPES) was designed under the Crum’s socio-critical conception of PE, contemplating integrated learning in the motor, cognitive, affective and social domains, to empower students to engage in significant PA, and actively participate in the movement culture throughout their lives [36]; expanding beyond a restricted and instrumental participation in PA [37]. Although the initial development of this syllabus slightly predates Whitehead’s influential works on PL [2], it implicitly aligns with the latter’s ontological and epistemological premises. Akin to a phenomenological and existentialist perspective [38], it advocates pedagogical practices of differentiation, allowing a high degree of flexibility towards the achievement of curricular goals, recognizing that each individual enjoys and values different forms of movement; while using assessment as a tool to motivate and identify where every student should work to improve, in line with strategies proposed both by PL [38] and assessment specialists [39].

The PPES distinguishes three learning areas: 1) Physical Activities, 2) Health-Related Fitness, 3) Knowledge. In the first area, it advocates the participation in a wide range of physical activities (sport-based team and individual activities, rhythmic and expressive activities, nature exploration activities, and traditional games), enabling students to choose from an eclectic array of physical activities throughout their life. In each of these activities, student progress is charted through 3 levels of competency – introductory, intermediate, and advanced – integrating 1) mastery of specific movement skills, 2) cognitive skills related to tactical decision, 3) knowledge and application of activity rules and 4) prosocial behavior during said activity [40]. This multilateral learning through participation in physical activities is supported by the development of health-related fitness, and the knowledge and skills needed to lead a healthy lifestyle through personal significant PA (second and third areas of the PPES, respectively).

Despite having common points with most PL definitions and models, the PPES curricular and pedagogical choices align more closely with the Australian proposal previously presented, since the latter explicitly includes the social domain as an integral part of the PL development, as well as elements pertaining to tactical and rules learning. Also, the APLF maps all development through the usage of a modified continuum based on the *Structure of Observed Learning Outcomes* taxonomy [23], which recognizes that learning might differ not only in quantity (i.e., being less or more skilled/knowledgeable) but in qualitative state as well (i.e., going from a descriptive, surface knowledge to a relational understanding of a skill/knowledge); a principle mirrored in the three levels of competency in the PPES.

Considering these specificities of the PPES design and implementation, none of the presented PL assessments provide a complete picture of learning in all four domains; nor were they designed for older adolescents. As such, we developed an instrumental system – *Portuguese Physical Literacy Assessment (PPLA)* – to address this gap, and use PE as a privileged mean for PL development in Portugal.

### The Portuguese physical literacy assessment (PPLA)

**Fig. 1:**
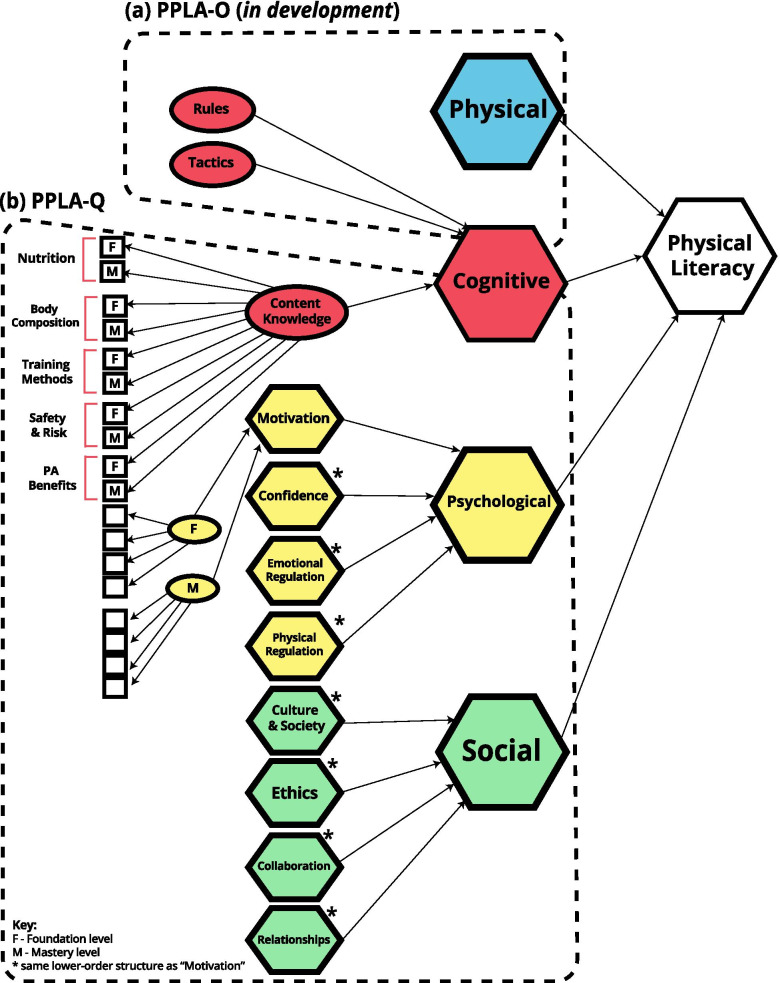
*Portuguese Physical Literacy Assessment* (PPLA) hypothesized model and instruments. Legend: PPLA is a tool comprised of two different instruments: **a** PPLA-Observation (PPLA-O) – assesses the physical domain, and the *Rules* and *Tactics* elements of the cognitive domain of PL; **b** PPLA-Questionnaire (PPLA-Q) – assesses the psychological, social and *Content Knowledge* element of the cognitive domain of PL

The PPLA was designed to provide a detailed and feasible assessment of each student’s PL journey, and to inform pedagogical decisions (at local, regional, and national level) towards a more meaningful and targeted environment to promote PL learning of grade 10–12 (15–18 years) adolescents. The PPLA (Fig. 1) is based on the PPES and integrates assessment in the four domains of the APLF, using two instruments: a) the PPLA-Observation (PPLA-O), and b) the PPLA-Questionnaire (PPLA-Q). The PPLA-O (still in development) uses observational data collected by the teachers during regular PE classes (competency levels in the different physical activities, and physical fitness levels using standardized protocols) to assess the physical and part (*Rules*, and *Tactics* elements) of the cognitive domain. The PPLA-Q, which will be the focus of this article, uses a knowledge test (with multiple-choice questions) and self-report (Likert-type scales) to assess the psychological, social and the remaining part of the cognitive domain (*Content Knowledge* element). Both these instruments were designed to be applied together to provide a holistic picture of each student’s PL journey.

PPLA (Fig. 1), following the APLF conceptualization of a learning continuum summarizes its five development levels (for each element of the four PL domains), into two learning levels: *Foundation* and *Mastery.* This simpler structure still captures the qualitative change in the learning experience, separating *surface learning* from *deep learning*, while providing a more parsimonious and feasible instrument.

The *Foundation* level represents the initial development of each element, building affective, cognitive, psychomotor and social structures that enable participation in movement and physical activities, albeit in an isolated, instrumental or externally focused manner (i.e., to obtain benefits/rewards, or conform to the norm) – akin to the *Unistructural* and *Multistructural* levels of the *Structure of Observed Learning Outcomes* taxonomy, and the foundational levels of Bloom’s Revised Affective Taxonomy [41].

*Mastery* level represents a deeper development of the element, invoking metacognitive processes, relational understanding, or internalized behaviors (i.e., integrated into the individual’s sense of self) regarding participating in movement and physical activities – derived from the *Relational* and *Extended Abstract* levels of *Structure of Observed Learning Outcomes* taxonomy, and higher levels in Bloom’s affective taxonomy.

As such, based on previous constructs studies of PL [29, 42] and the structure implied by the APLF, we hypothesize a hierarchical measurement model, with PL conceptualized as a fourth-order formative construct (Fig. 1) composed by its four domains (third-order formative constructs). Each domain is then formatively composed by several elements (second-order formative constructs), in turn composed by two first-order constructs, reflexively formed by a set of manifest indicators (i.e., items).

The distinction between *formative* constructs (i.e., composites) and *reflexive* constructs (i.e., factors) is important here. While the later assumes that items (or lower-order constructs) are interchangeable – since they measure the same underlying trait (i.e. are unidimensional) – and thus are expected to covary, the former assumes the opposite: that its composing items are not interchangeable, and are not expected to covary – where an omission or deletion of an item changes the essence of the construct being measured [43–45].

Based on this conceptual framework, in a series of studies, we sought to develop the *PPLA-Questionnaire (PPLA-Q)*, an instrument comprised of modules to assess grade 10–12 adolescents’ *psychological*, *social* and part of *cognitive* domains of PL; and gather evidence for its content validity, feasibility within PE setting, preliminary reliability, item difficulty and discrimination.

## Methods

### Studies overview

**Fig. 2:**
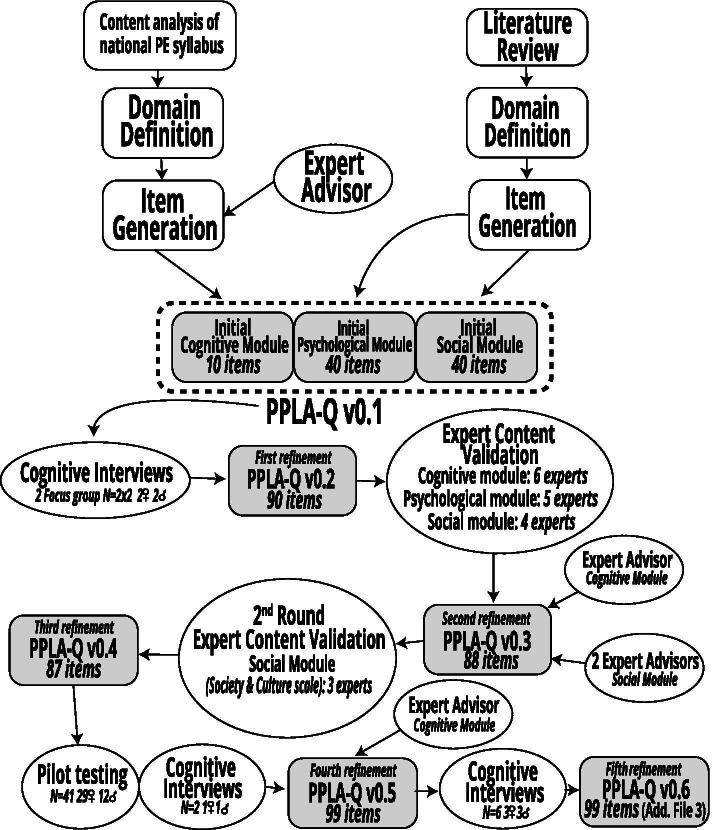
Overview of development studies of the *Portuguese Physical Literacy Assessment – Questionnaire* (PPLA-Q)

The development of the *Portuguese Physical Literacy Assessment Questionnaire* (PPLA-Q) entailed a series of studies (Fig. 2), based on a multiple phase design [46–48], inspired by the psychological, social and cognitive domains of the PL model proposed in the APLF [1, 49], and by the Portuguese PE syllabus [50–52].

All the work was done in Portugal, as part of the doctoral project of the lead author, approved by the Ethics Council of Faculty of Human Kinetics, as well as the Portuguese Directorate-General of Education. All methods were performed in accordance with the relevant guidelines and regulations.

PPLA initial development was based on previous work done in the Erasmus+ Sport Project: PhyLit – Physical Literacy (590844-EPP-1-2017-1-UK-SPO-SSCP, January– December 2018), where a panel of experts selected – among the 30 proposed by the APLF – relevant elements for developing and advocating PL as an essential competence for European citizenship, based on a literature review of existing conceptualizations [19].

Initial development for each of the three modules of PPLA-Q entailed domain identification and item generation; followed by an iterative process to gather judgmental evidence on content validity that included: two rounds of qualitative and quantitative expert validation; three rounds of cognitive interviews with high-school students; and multiple instances of expert advisor input. We also conducted a pilot study to assess feasibility of the questionnaire in PE and collect preliminary data on reliability and construct validity.

### Domain identification

Based on literature review, we established a theoretical framework for each of the eight elements in the psychological (*Motivation, Confidence, Emotional Regulation,* and *Physical Regulation)* and social domains (*Culture & Society, Ethics, Collaboration,* and *Relationships*) (Table 1). The literature review conducted by Dudley and colleagues [49], in the report preceding the creation of the APLF, was used as starting point to identify established and relevant theories for each element in the literature of motor development, physical education and/or physical activity. Then, constructs with higher conceptual proximity were chosen – caring to minimize overlap –, mapped to the two-level framework, and operational definitions derived from the APLF.

**Table 1:** Domain identification for the psychological and social domains

	Theoretical framework	Operational definition (number of items)	Instruments used as reference
**Psychological Domain**			
**Motivation**	Self-determination Theory [53, 54]	**Reasons for engaging in movement and physical activity in response to internal or external factors** [1]	Behavioral Regulation in Exercise Questionnaire – 3 (BREQ-3) [55, 56]
		Foundation: Controlled motivation (5 items)	
		Mastery: Autonomous motivation (5 items)	
**Confidence**	Psychological need satisfaction -Perceived competence [57]	**A belief in self-worth and ability to perform in movement and physical activity** [1]	Psychological Need Satisfaction in Exercise Scale (PNSE) [56]
		Foundation: Beliefs of self-worth and ability (5 items)	
		Mastery: Beliefs of self-worth and ability in challenging contexts (5 items)	
**Emotional Regulation**	Emotional Intelligence [58]	**Ability to manage emotions and resulting behaviors in relation to movement and physical activity** [1]	Wong and Law’s Emotional Intelligence Scale (WLEIS) [59]
		Foundation: Awareness of own emotions and other’s (5 items)	
		Mastery: Emotional regulation and control (5 items)	
**Physical Regulation**	NA	**Recognizing and managing physical signals such as pain, fatigue and exertion** [1]	NA
		Foundation: Awareness of physical signals (5 items)	
		Mastery: Regulation and management of physical signals (5 items)	
**Social Domain**			
**Culture & Society**	Sport Education [60]	**Appreciation of cultural values which exist within groups, organizations and communites** [1]	NA
		Foundation: Participation in sport’s cultural phenomena (5 items)	
		Mastery: Valuing participation in sport’s cultural phenomena and encouragement of others to do so (5 items)	
**Ethics**	Moral development [61, 62]	**Moral principles that govern a person’s behavior, relating to fairness and justice, inclusion, equity, integrity, and respect** [1]	Fair Play Questionnaire in Physical Education (FPQ-PE) [63]
		Foundation: Respect for basic moral and ethical principles in physical activity contexts (fair-play) (5 items)	
		Mastery: Autonomy and empowerment of others in respecting moral and ethical principles in physical activity contexts (fair-play) (5 items)	
**Collaboration**	Personal and Social Responsibility [64]	**Social skills for successful interaction with others, including: communication, cooperation, leadership and conflict resolution** [1]	Personal and Social Responsability Questionnaire (PSRQ) [65]
		Foundation: Respect and cooperation with others	
		Mastery: Caring and leading others to success	
**Relationships**	Psychological need satisfaction -Perceived Relatedness [57]	**Building and maintaining respectful relationships that enable a person to interact effectively with others** [1].	Psychological Need Satisfaction in Exercise Scale (PNSE) [56]
		Foundation: Interaction and relatedness with others	
		Mastery: Management and maintaining relationships with others	

For the Cognitive Domain, we conducted a content analysis of the Portuguese PE syllabus (PPES) to identify key learning objectives coherent with the *Content Knowledge*, *Tactics* and *Rules* elements of the APLF. In this process, to ensure adequate content representation, we subdivided the *Content Knowledge* element into different content themes (*Nutrition, Body Composition, Training Methods, Safety & Risk, PA Benefits);* each was then mapped to the two-level framework and its operational definition derived from the PPES (Table 2).

**Table 2:** Domain identification for the cognitive domain

Content	Operational definition
**Nutrition**	Foundation: Identify healthy food options (C1)
	Mastery: Evaluate impact of energetical balance in regulation of body weight (C2)
**Fitness and training**	Foundation: Identify main components of physical fitness (C3)
	Mastery: Evaluate training methods for components of physical fitness (C4)
**Safety and risk**	Foundation: Identify safety rules and principles in physical activities (C5)
	Mastery: Interpret doping’s impact on health and sport ethics (C6)
**PA Health Benefits**	Foundation: Identify general physical activity guidelines for children, adolescents, and adults^a^(C7)
	Mastery: Relate types of training with their benefits for health (C8)
**Body composition**	Foundation: Identify Body Mass Index’s calculation formula (C9)
	Mastery: Evaluate body composition profile and make recommendations (C10)

*PA* Physical Activity

^a^According to World Health Organization [66]

Since tactical behaviors and adherence to rules (i.e., as a participant, and as a referee or judge) are better assessed through direct observation of the student’s behavior during PE, we chose to include the *Tactics* and *Rules* elements alongside the assessment of the physical domain (in the PPLA-O). As such, these elements will not be further discussed here, despite them being integral part of the Cognitive domain.

### Item generation

#### Psychological and social modules

Items in the Psychological and Social domains were developed to conform to self-report measurement using Likert-type scaling, given its adequacy and versatility to measure attitudes, beliefs and self-perceived abilities [67, 68]. An initial goal was set to generate a 5-item subscale per learning level (two subscales per element, four elements per module). This was a compromise between the size of the resulting questionnaire, and a larger initial item pool to provide margin for eliminating poorly performing items during testing [67, 69]; down to four per subscale – the recommended number to calculate reliability and further test measurement models [70].

In an effort to use psychometrically sound items as a reference for item generation [71] a non-systematic literature review was conducted using ERIC, Google Scholar, Scopus and ProQuest databases to identify a first round of eligible articles for each element, which were then used to refine further searches for articles. In these, we selected published and validated scales or subscales (in English or Portuguese), amply used in PE, sport, or PA contexts, and sampled items that adhered to each level’s operational definitions (Table 1). When various identical items overlapped in content, those with higher item loading were selected.

After permission for adaptation was granted by each scale’s lead author, sampled items were used as reference to generate items in Portuguese, based on the examples provided by the APLF, and technical recommendations available in the literature [67–69, 72, 73]. When suitable reference scales were not available or failed to achieve full content representation for the element, or level, items were generated according to previous literature view.

All items used a consistent 5-points unipolar response scale, to maximize reliability and validity [73, 74] . Response points were fully labelled, using both numeric and verbal labels, (0 = *Not at all*; 1 = *Slightly*; 2 = *Moderately*; 3 = *Quite a lot*; 4 = *Totally*), measuring student’s identification with each of the statements (*How much do the following statements describe you?)*.

#### Cognitive domain

For their suitability to test cognitive ability and knowledge [68], and ease of application, multiple-choice questions were generated for each content theme and level (10 items), according to technical advice presented by the literature [75, 76], and by an educational assessment expert (PhD holder with extensive experience as a PE and graduate-level college professor, as well as an employee in the Portuguese Institute for Educational Assessment).

Throughout the process in all modules, the lead author acted as item generator, while remaining authors acted as co-validators to ensure preliminary content validity.

### Content validity

Content validity pertains to the extent to which a set of items represents the intended construct [67]. It requires evidence of content relevance, representativeness, and technical quality, assessed through evaluation by experts and population judges [47]. As such, we led an iterative process with multiple rounds [77], collecting both qualitative and quantitative evidence from both parties.

#### Cognitive interviews

Cognitive interviewing is a qualitative method to assess whether a survey fulfills its intended purpose, through interview of selected individuals, before, during and after pretesting [78]. In our study, cognitive interviews were conducted in three rounds, in two different high schools in Lisbon – one with a dominantly higher socioeconomic status population, and another with a lower socioeconomic status population – involving students of the target age-group (15–18 years), through different phases of development of the PPLA-Q. Before participation, informed consent was provided by all students and their legal guardians. All interviews were conducted by the lead author during PE classes and recorded. Initial interviews were more extensive (i.e., more content, less depth), while the latter ones were progressively more intensive (i.e., narrower content, higher depth). This strategy balanced gross evaluation (e.g., format, conceptual breadth) in earlier phases with fine-tuning (e.g., wording, syntax) in later ones.

In February 2020, in each high school, a cognitive interview was conducted with a group of two students from grade 10 (aged 15) and another with two grade 11 students (aged 17). We sought to diversify these groups by 1) including, in each, a female, and a male, with different PE competency levels (according to their teacher); and 2) including students from different majors: one group from a Science, Technology, Engineering and Math major, the other from a Humanities and Arts major. Students were asked to fill in a draft version of the PPLA-Q, marking any items with ambiguous or unclear wording. Afterwards, an interview was conducted to probe for comprehension of items – focusing on the ones marked by students. Students were asked to verbally express their understanding of each and paraphrase it according to their own words. They were also questioned about general issues of the questionnaire (i.e., length and structure, layout, ease of reading, rating scales, comprehension of instructions and item stems). Average duration was 45 min.

In December 2020, a second round of individual cognitive interviews was conducted immediately after pilot testing (version 0.4 of PPLA-Q) with two students from grade 10 (1 female, 1 male, both aged 15) from a Humanities major class. Here, students who posed abundant questions during the questionnaire application were selected to better study the clarity of the items. Given time constraints of the project, this round enlisted less students that initially warranted. Students were asked about their comprehension of selected items – those which were the target of most of student’s questions during pilot testing, as well as those previously revised. Average duration was 17 min.

In January 2021, a third round of individual cognitive interviews was conducted with six different students from the same grade 10 Humanities class recruited for last round (3 female, 3 males, mean age = 14.8 years). These were selected according to as different PE competency levels as possible (reported by the teacher). They were asked about their comprehension of all items changed from version 0.4 to version 0.5. Average duration was 15 min.

#### Evaluation by experts

Among the many methods available, Content Validity Index (CVI) and Cohen’s coefficient *kappa* (**κ**) for interrater agreement were used to systematically assess expert consensus on content validity of an instrument [47, 79].

Given different subject matter for each of the modules, expert selection was stratified per module to allow for more useful inferences. We intended to collect evidence from 6 experts – following recommendations of 5 [80] - with relevant scientific and professional background, on each of the questionnaire’s domains (i.e., psychology of physical activities/sport; sociology of sport; educational assessment/curriculum development), and ideally with experience in instrument development [81]. According to their expertise, each expert was invited to participate either (a) in all 3 modules (*n* = 3); (b) in 2 modules (*n* = 1) or (c) in a single module (*n* = 11).

Experts were invited through an email presenting the project’s goals and explaining the motives for selection, containing (1) instructions for intended contribution, (2) a draft version of PPLA-Q, and (3) a spreadsheet file. Operational definitions for each construct were also provided – as content validity is inextricably linked to the definition of constructs under examination [67]. In the spreadsheet file, experts were asked to: (1) rate each item on its relevance (“*How important is the item to assess the targeted construct*?”) and clarity (“*Is the wording of the item clear*?”), (2) provide suggestions for item improvement, (3) provide suggestions on questionnaire structure, instructions, and rating scale. Both relevance and clarity were assessed with a 4-point Likert-type Scale [80]. For relevance the rating options were: 1 = *not relevant*, 2 = *somewhat relevant*, 3 = *quite relevant*, and 4 = *very relevan*t [79]. For clarity, the options were: 1 = *not clear*, 2 = *item needs revision*, 3 = *clear, but needs minor revision*, 4 = *very clear* [82]. During analysis, both ratings were collapsed into two dichotomous categories (“content invalid” and “not clear” for ratings of 1 and 2, and “content valid” and “clear” for ratings of 3 and 4, respectively) [80].

Of the invited experts, the actual first-round expert sample (*n* = 10) consisted of 2 global experts (3 modules), 1 expert rating 2 modules, and 7 experts rating a single module. Another expert provided solely qualitative feedback (i.e., suggestions of improvements for item and questionnaire structure) on 2 of the modules, with no quantitative ratings. We had minimal missing data, with no bearing on calculations, since all adjusted for the total number of raters in each item. Further characteristics about the participating experts are summed up in Additional file 1.

All calculations used *RStudio* [83] with *R* version 4.0.2 [84]. CVI was computed both at item level (I-CVI) and module level (S-CVI/Ave and S-CVI/UA). Polit & Beck [85] argue that given diverse uses of CVI in the literature, one should explicit their calculations. We computed I-CVI as the proportion of experts rating each item as *content valid*. S-CVI/Ave was computed as the average of I-CVI for each module, while S-CVI/UA was computed as the proportion of items with I-CVI = 1 (i.e., *universal agreement*) for each module.

Many authors have criticized drawing content validity evidence based solely on CVI, given its susceptibility to chance agreement. They propose that Cohen’s *kappa* [86] – a statistic which accounts for the possibility of chance agreement of experts – be used alongside CVI [79]. For this purpose, *kappa* (**κ**) was computed using Fleiss’s modified version for multiple raters [77, 87] for each item:

$$k=\frac{\left(\ {P}_a-{P}_c\right)}{\left(1-{P}_c\right)}$$

where P_a_ (proportion of agreement) = *I-CVI* for the item, and where *P*_*c*_ (probability of a chance agreement), for a random binomial variable, with one outcome:

$${P}_c=\left(\frac{N!}{A!\left(N-A\right)!}\right)\ast {.5}^N$$

With N = number of experts, and A = number of experts rating item as content valid.

For item clarity, an identical procedure was used to calculate proportion of agreement (akin to I-CVI), and a ***κ*** statistic for each item. as the usual application of Content Validity Index (CVI) pertains to a global evaluation of the item [77], which might hide some crucial aspects of the item’s quality, confounding the conceptual relevance of the item, with the clarity of its wording.

We used ***κ*** to inform item level decisions, evaluating item relevance as fair (.40 to .59), good (.60 to .74) and excellent (> .74); **κ** lower than .40 prompted elimination of the item [87, 88]. For clarity, the threshold increased to discriminate items needing minor revisions and ensure higher clarity throughout: we evaluated items as clear (**κ** > .74) and as needing revision (**κ** < .74).

Scale level decisions were informed by S-CVI. We used literature recommendation of .80 as an adequate level of agreement for the more stringent S-CVI/UA [81], and .90 for S-CVI/Ave [89].

In the second round of expert evaluation, the same procedures were followed to gather evidence of content validity on the revised *Culture & Society* scale (version 0.3), targeting a lower number of experts (*n* = 3, 2 of which participated in the previous round), due to time constraints in the project schedule.

### Pilot testing

Pilot testing, or *pretesting* constitutes an opportunity to (1) test the application of items in development to a representative sample of target population [90]; (2) gather feasibility evidence to plan a larger scale study [91]; and (3) gather data for preliminary item analysis and estimates of reliability [92].

Although no clear-cut standard is available for sample size of pilot tests, Hertzog [91] suggests a sample size of 40 individuals for estimating preliminary data on reliability and item discrimination. As such, we pilot tested version 0.4 of PPLA-Q with a sample of 41 grade 10 students (down from an initial pool of 58 students who received the informed consent), from two classes of the different schools in Lisbon (n_school1_ = 19, n_school2_ = 22) aforementioned – one with a higher socioeconomic status population, another with a lower socioeconomic status population, as attested in each school’s pedagogical project. This sample was composed of 29 females (71%) and had an average age of 15 (0.4) years. All students provided an informed consent signed by themselves and their legal guardian.

PPLA-Q was self-administered, in pen and paper format, both in PE gym and classroom setting – to test likely settings expected for future application – in presence of the lead author. Students were instructed to state any question regarding questionnaire’s instruction, items, or rating scales. Application was timed to calculate average completion time; attrition rate was calculated as the percentage of students completing the study, among those who received the informed consent.

#### Preliminary item analysis

##### Psychological and social modules

Given the novel status of any construct validation under the APLF model, as well as a complex and high number of constructs under analysis, we chose to conduct preliminary item analysis using the partial least squares – structural equations modelling (PLS-SEM) framework [93]*.* No a priori power analysis was conducted, since our goal was to gather very rough insights into the statistical behavior of the measurement model of items. Despite this, our sample size approximated the thumb-rule of 10 times the maximum number of indicators per construct [44]*.*

Prior to calculation, data was scanned for suspicious response patterns, and items P1 to P5 were reversed-scored – since they refer to *controlled motivation,* and thus expected to negatively load on the second-order motivation construct. Missing data was below the 5% threshold for every indicator (i.e., item), under which circumstances PLS-SEM is robust [44]. *SmartPLS* 3.2 [94] was used to calculate Cronbach’s **α**, composite reliability and outer loadings (factor weighting scheme, with 300 iterations and stop criterion of 1*10^− 7^) using a *Hierarchical Component Model* (reflective-formative) for each of the modules, with the repeated-indicator approach [44, 95].

For interpretation, we followed Hair’s et al. [44] advice of using both **α** and composite reliability – as lower bound and upper bound estimates of reliability, respectively. **α** was deemed acceptable at .70 [96, 97], while composite reliability was deemed acceptable at .60 [44]. As for indicator reliability (outer loadings) values of .70 were deemed acceptable [44].

##### Cognitive module

In order to gather preliminary evidence on construct validity for items in the cognitive module, we analyzed item’s difficulty index, discrimination index, and performed a distractor analysis [75, 89] under the Classical Test Theory framework.

We had missing data for one student who did not complete this module. Item were scored using the *CTT* package [98] in *RStudio* [83] with *R* version 4.0.2 [84]; we used dichotomous scoring (i.e., 0 and 1) for correct answers – multiple selection items were considered correct if all correct options were selected. Difficulty and discrimination (*gULI*) indexes calculation, and distractor analysis (proportion of responses in each distractor) were calculated with the *shinyItemAnalysis* package [99].

Item discrimination was interpreted according to cut-offs of *Very good* (>.40); *Reasonably good (*.30–.39); *Marginal (*.20–.29), *Poor (*<.19) [100, 101]. Distractors with lower than 10% of responses were considered poor functioning, to impose a stricter quality standard, although a lenient threshold of 5% is usually proposed [102].

## Results

The following sections are organized chronologically, as to provide the reader with a detailed view of the different development phases and refinements that the PPLA-Q went through. In the Discussion section, we summarize and discuss these results according to their overarching goal (e.g., content validity).

### Domain identification

#### Psychological domain

##### Motivation

Self-Determination Theory (SDT) [53] has abundant research in exercise and physical activity contexts [103]. One of its mini-theories, Organismic Integration theory [104], posits a continuum of different behavioral regulations varying according to their degree of self-determination. Among these, *external* and *introjected* are posited as more *controlled* (i.e., less autonomous) forms of extrinsic motivation; while *identified*, *integrated* and *intrinsic* are posited as more *autonomous* forms of motivation. More autonomous forms have shown positive association with increased participation in PA [105], and with positive experiences in PE [106]. We placed controlled forms of motivation in the foundational level, and more autonomous forms into the mastery level – following a two factor structure proposed in previous research [107].

##### Confidence

Multiple self-concept constructs in the literature center around the belief in one’s abilities to perform in PA settings; of these, (perceived) competence and self-efficacy seem to be determinants of participation in PA in children and adolescents [105, 108]*.* Although conceptualized under different frameworks – perceived competence in the SDT tradition (as a basic psychological need driving motivation), and self-efficacy as the main construct of Social-Cognitive Theory (SCT) [109] – studies have called for their integration, since they stem from the same concept of human agency [110], and might share a common core [111]*.* As such, we integrated perceived competence – given its centrality to SDT, and similarity to task self-efficacy – in the foundation level, and barrier self-efficacy [112] (i.e., belief in one’s ability under challenging conditions) in the mastery level.

##### Emotional regulation

Self-regulation is a broad concept that entails the individual’s capacity to override and alter their behavior towards a standard or goal [113]*.* When referring to the affective domain, the construct of Emotional intelligence (i.e., ability to perceive and regulate emotion) [58, 114] has gained visible traction in research. It has been linked to PA participation, both as an outcome and as predictor [115]*.* Among its many conceptualizations we chose to adapt Wong and Law’s Emotional Intelligence Scale’s factorial approach [59], mapping emotional evaluation (own and interpersonal) to the foundation level, and use and regulation of emotions to the mastery level.

##### Physical regulation

Although we failed to identify a PA-specific construct that dealt with APLF’s idea of regulating physiological signals and effort during PA– analogous to emotional regulation - we found it related to other affective constructs such as activity pacing (i.e., regulation of activity level towards an adaptive goal) [116] and coping (i.e., behavioral and cognitive efforts to manage internal and external demands during stressful situations) [117]*.* The latter has been researched mainly in performance-oriented settings, and has showed positive association with sport commitment in adolescents [118]*.* As such, we integrated this concept in an identical structure to that of Emotional Regulation: perception of changes in the body during exercise in the foundational level; and regulation of effort in the mastery level.

#### Social domain

##### Culture & Society

The Culture & Society element is defined in the APLF as the appreciation of values present within communities of PA practice, however, we argue that its operationalization deals with cultural tolerance and cultural intelligence [119], rather than with the specific participation and appreciation of the cultural phenomenon of sport and PA. As such, we based this construct on Siedentop’s call for symbolic attributes like values, rituals and traditions to be an integral part of PL [60]*.* This ritualist facet manifests through the use of specific attire, jargon, and participation in select behaviors and habits [120]; as well as through displays of fandom and sport fan passion [121]*.* All these further contribute to feelings of affiliation and membership in a collective identity [122]; and although literature linking this phenomena to participation in PA is sparse, it is plausible that it might play a mediator role in increasing perceived relatedness [123], and emotional regulation – particularly in anxiety-inducing settings [124]*.* We chose to map participation in cultural behaviors to the foundational level, while the mastery level represents a more involved stance in these (i.e., valuing and encouraging participation).

##### Ethics

Fair play, is an integral part of modern sport as its major ethical system – coherent with universal values [125, 126]*.* PE plays a critical role in teaching this “inner morality of sport”, which surpasses simple adherence to rules, and includes following unwritten rules and moral codes [126]*.* Interiorization of these moral codes are concomitant with mature stages of moral development, which are known antecedents of prosocial behavior [61] (i.e., acts involving care for welfare of others) [127], and might also increase intrinsic motivation in PA settings [128, 129]*.* We chose to use Gibbs’ [61] model of moral development which, based on Kohlberg’s work [62], identifies two main levels in standard moral development: immature (i.e., a pragmatic, instrumental sense of morality, mapped to the foundational levels) and mature (i.e., based on social values and empathy, mapped to the mastery levels).

##### Collaboration

Personal and social responsibility are the main focus of Hellison’s [64] *Teaching Personal and Social Responsibility* (TPSR) model for developing prosocial behavior, providing a way to address holistic development of students in PE, and enable them with life skills for active citizenship through five levels: (1) Respect for the rights and feeling of others, (2) Effort and cooperation, (3) Self-direction, (4) Caring and helping others, (5) Transfer outside the gym. Evidence shows association of its application with many positive emotional, psychological, and social outcomes (e.g., self-efficacy, self-regulation, caring, conflict resolution) [130]*.* It is also suggested that students’ level of personal and social responsibility are associated with intrinsic motivation in PE [65]*.* To avoid overlap between personal responsibility and other elements tapping into similar concepts (i.e., Ethics, Emotional and Physical regulation), we mapped TPSR’s “Respect” level into the foundational level, and “Caring and Helping” into the mastery one, based on the works of Li’s et al. [65].

##### Relationships

Relatedness (i.e., perceived connection with others) is another one of the basic psychological needs posited to drive motivation according to SDT. Despite its theoretical relevance, evidence has shown little to no direct association between relatedness and participation in PA, in both general [103, 105] and PE contexts [131]*.* However, some authors [103, 132] suggest that this might be due to relatedness being highly context-dependent (i.e., affected by prevalence of solitary exercise, or lack of connection with classmates), and thus, not captured in its entirety in the researched contexts. This idea is further reinforced by evidence of peer-support associating with PA practice [133], positive outcomes in PE [106], and as mediator in other relevant outcomes as effort [134] and enjoyment [132]*.* In our model, akin to Collaboration, we mapped a reactive role in relationships to the foundational level, while the mastery level presupposes an active role in relationship development.

#### Cognitive domain

##### Content knowledge

Few studies have examined the relationship between knowledge regarding PA, and outcomes in PE contexts (either affective, social, or behavioral). However, there is evidence of positive association of knowledge of PA guidelines [66] and health benefits, both with PA participation in young adults [135, 136], and physical fitness [137]. Similarly, awareness of health risks related to inactivity might predict PA participation in adults [138] and adolescents [139]. A consensus among aforementioned studies seems to be that knowledge of these contents is consistently low, with similar evidence in Portugal: both in PE setting [140] and in young adults [141].

### Content validity

#### Version one (v0.1): cognitive interviews

All students (*n* = 4) referred to the questionnaire as having an adequate layout and length, as well as clear directions for filling in the questionnaire. Their understating of item stems and rating scales, in the psychological and social modules, matched our intention: with equivalent conceptual distance between rating scale options. The response options in the cognitive module were deemed intuitive, given their familiarity with multiple-choice items. Item content was mostly clear for all students, with some difficulties arising in discerning the meaning of many items in the *Culture & Society* scale; they suggested adding examples to clarify concepts like “cultural diversity” and “traditional physical activities”.

We found a quality issue with the cognitive module item C6 (i.e., doping’s impact on health and fair play): During think-aloud response, it became evident that students could extrapolate the correct response without pertinent knowledge, due to the implausibility of distractors. According to students’ comments changes were made to the questionnaire: we added examples for mentioned concepts and improved the plausibility of C6’s distractors.

#### Version two (v0.2): expert evaluation – 1st round

To quantitatively assess the *relevance* and *clarity* of each item, a panel of subject matter experts were asked to rate each item on a 4-point Likert-type scale. Ten experts in total participated in this round, of these 6,5 and 4 experts rated the cognitive, psychological, and social modules, respectively. Based on their ratings, CVI (I-CVI, S-CVI/Ave, and SCI/UA) and **κ** were calculated.

##### Item relevance

Item CVI ranged from .33 to 1 (cf. Additional file 2): 1 item had a CVI of .33, 3 items had a CVI of .5, 10 items had a CVI of .75, 6 items had a CVI of .8 and the remaining 70 items a CVI of 1.

**Κ** ranged from .13 to 1, with 86 items (96%) considered either excellent (76 items) or good (10 items) (Table 3); four items were prompted for elimination – one in the cognitive module (C2, *Nutrition*) and three in the social module (S3 in *Culture & Society* scale, and S30 and S34 in *Relationships* scale) (Table 3).

**Table 3:** Number of items, per scale, in each kappa category of relevance and clarity in result of expert evaluation (version 0.2 and 0.3)

Module Element (items)	Relevance				Clarity	
	Kappa^**1**^			S-CVI (Ave^**2**^/UA^**3**^)	Kappa^**4**^	
	Elimination	Good	Excellent		Revision	Clear
**1st round (version 0.2)**						
**Cognitive**				**.87 / .60**		
Nutrition (C1 & C2)	1	–	1		1	1
Fitness and training (C3 & C4)	–	–	2			2
Safety and risk (C5 & C6)	–	–	2		1	1
Health benefits of PA (C7 & C8)	–	–	2		1	1
Body composition (C9 & C10)	–	–	2		–	2
**Psychological**				**.98 / .93**		
Motivation (P1-P9, P37)	–	–	10		1	9
Confidence (P10-P18, P38)	–	–	10		–	10
Emotional Regulation (P19-P27, P39)	–	–	10		–	10
Physical Regulation (P28-P36, P40)	–	–	10		1	9
**Social**				**.90 / .68**		
Culture & Society (S1-S9, S37)	1	4	5		4	6
Ethics (S10-S18, S38)	–	–	10		–	10
Collaboration (S19-S27, S39)	–	4	6		3	7
Relationships (S28-S36, S40)	2	1	7		2	8
**2nd round (version 0.3)**						
**Social**				.96* / .84*		
Culture & Society	–	–	10		5	5

^1^Multirater modified kappa designating agreement on relevance: **κ** = (I-CVI - pc)/(1 -pc), with pc (probability of a chance occurrence) computed using the formula for a binomial random variable, with one specific outcome [77];evaluation criteria for kappa [87, 88]: Elimination <.40, Fair kappa of .40 to .59; Good kappa .60 to .74; and Excellent kappa > .74

^2^ S-SCI/ Ave - Scale CVI Average: Calculated by averaging all I-CVI in scale/module

^3^ S-SCI/ UA - Scale CVI Universal Agreement: Calculated by dividing the sum of items with I-CVI of 1.0 by module’s total number of items

^4^Modified criteria for kappa: Needs Revision < .74; Clear > .74

*Calculation included all scales of social module

##### Scale relevance

The psychological and social modules showed adequate content validity, with a S-CVI/Ave of .98 and .90 respectively [89]; while the cognitive module failed to reach the proposed adequacy threshold of .90, with an S-CVI/Ave of .87.

According to the S-CVI/UA, only the psychological module showed adequate content validity, with a value of .93 (higher than the .80 threshold) [81], while the cognitive and social modules did not - .60, and .68 respectively.

##### Item clarity

Proportion of agreement ranged from .33 to 1 (cf. Additional file 2): 2 items had an index of .33, 2 items had an index of .50, 2 had an index of .67, 8 items had an index of .75, 7 items had an index of .80, and the remaining 69 items an index of 1.

**Κ** for clarity ranged from −.07 to 1 (Additional file 2) with 76 items (84%) considered clear and 14 items prompted for revision – the *Culture & Society* scale had the greatest number of items needing revision, followed by *Collaboration* and *Relationships*, all in the social module.

##### Questionnaire refinement

Based on both qualitative and quantitative evidence from experts, two items were eliminated from the Relationships scale. It also prompted a major revision of the *Culture & Society* scale to increase S-CVI/Ave and S-CVI/UA of the social module to acceptable levels – informed by consultation with a subject matter expert, and one of APLF’s authors.

The cognitive module underwent restructuration as most experts commented on quality issues regarding (1) implausibility of distractors, (2) syntax and (3) structure. None of the items were eliminated, as it would compromise content representation, and the two-level framework of the module. Albeit not reaching the desired threshold for S-CVI (Ave and UA), we chose not to submit the cognitive module to a formal second round of expert evaluation, given that all **κ’**s (relevance) were excellent (>.74), save from item C2. Alternatively, we consulted with an assessment expert to restructure item C2 and improve the clarity on items C6 and C8, with no changes content-wise.

#### Version three (v0.3): 2nd round results for Culture & Society element

We asked 3 experts to participate in a second round of evaluation of *Culture & Society* scale, given the depth of its restructuration. Same procedures and calculations applied from the 1st round.

All items in the revised *Culture & Society* scale obtained a I-CVI and **κ** of 1, indicating absolute agreement on item’s relevance (Additional file 2). As such, S-CVI/UA of the social module increased to .84, entering an acceptable range [81].

Proportion of agreement on clarity ranged from .33 to 1 (Additional file 2): 1 item with .33, 4 items with .67 and the remaining 4 with 1; **κ** ranged from −.07 to 1, with 5 items considered clear and 5 prompted for revision.

##### Questionnaire refinement

Five items in the *Culture & Society* scale – with clarity **κ** lower than .74 – were revised (S3 – S5, S7 and S9), and S6 was eliminated, since expert’s comments pointed to it being more representative of general cultural tolerance than adherence to sport’s culture.

### Version four (v0.4): pilot Testing & Cognitive Interviews

#### Feasibility

Of the 58 students who got the informed consent, 41 completed the PPLA-Q, resulting in an attrition rate of about 30%. These 41 students (71% female) studied in grade 10 of two different schools, with two different majors (19 students from a Science, Technology, Engineering and Math course, 22 from Humanistic course), mean age 15 (0.4) years.

Completion time was gathered to assess the questionnaire’s feasibility during PE classes. Average completion time was 27 (7) minutes (*n* = 34, with the remaining 7 students failing to fill in the beginning and ending time). Questionnaire application in the gym allowed for ample space between students, which restricted talking; however, application in a crowded classroom promoted student’s sharing ideas about the items, and their correct option(s) (in the cognitive module). No response errors or any suspicious response patterns were identified on the responses (e.g., straight or diagonal lining, or alternating poles) [44].

#### Preliminary reliability (psychological and social modules)

Preliminary reliability for each subscale, as well as each item’s outer loading (indicator reliability) on its intended construct are summarized in Table 4.

**Table 4:** Preliminary item and subscale reliability of Psychological and Social modules (*n* = 41; PPLA-Q version 0.4)

Psychological Module				Social Module			
Element (Subscale)	Item	Outer Loading	Subscale Reliability^**a**^	Element (Subscale)	Item	Outer Loading	Subscale Reliability^**a**^
**Motivation** (Foundation)	P1	.32	**.76**/ .22	**Culture** (Foundation)	S1	.69	.66/ **.79**
	P2	−.81			S2	**.79**	
	P3	−.77			S3	.34	
	P4	.37			S4	**.91**	
	P5	−.10					
**Motivation** (Mastery)	P6	**.85**	**.87/ .91**	**Culture** (Mastery)	S5	**.88**	**.86/ .90**
	P7	**.86**			S6	**.87**	
	P8	**.88**			S7	**.81**	
	P9	**.80**			S8	**.89**	
	P37	.67			S34	.54	
**Confidence** (Foundation)	P10	**.88**	.**93/ .95**	**Ethics** (Foundation)	S9	−.14	.57/ .59
	P11	**.87**			S10	−.95	
	P12	**.92**			S11	−.88	
	P13	**.91**			S12	−.28	
	P14	**.86**			S13	.11	
**Confidence** (Mastery)	P15	**.81**	**.70**/ **.80**	**Ethics** (Mastery)	S14	.27	.36/ .53
	P16	**.84**			S15	**.81**	
	P17	.21			S16	−.51	
	P18	**.81**			S17	**.72**	
	P38	.59			S35	.61	
**Emotional Regulation** (Foundation)	P19	**.70**	**.75/ .76**	**Collaboration** (Foundation)	S18	**.77**	**.81/ .87**
	P20	.66			S19	**.91**	
	P21	.44			S20	.58	
	P22	**.74**			S21	.69	
	P23	.55			S22	**.80**	
**Emotional Regulation** (Mastery)	P24	.68	.61/ **.78**	**Collaboration** (Mastery)	S23	**.83**	**.80/ .87**
	P25	**.75**			S24	**.84**	
	P26	.65			S25	**.88**	
	P27	**.85**			S26	**.80**	
	P39	.21			S36	.35	
**Physical Regulation** (Foundation)	P28	−.28	.62/ .17	**Relationships** (Foundation)	S27	**.73**	.**85/ .90**
	P29	−.26			S28	**.79**	
	P30	−.14			S29	**.90**	
	P31	**.78**			S30	**.91**	
	P32	**.76**					
**Physical Regulation** (Mastery)	P33	**.80**	**.76/ .84**	**Relationships** (Mastery)	S31	**.91**	**.74/ .91**
	P34	.38			S32	.68	
	P35	**.85**			S33	**.79**	
	P36	**.83**			S37	.60	
	P40	.69					

^a^Statistics presented: Cronbach’s **α** / Composite Reliability

Note: Results higher than .70 (outer loading and **α**) and .60 (composite reliability) are bolded (acceptability threshold)

Ten of the subscales (63%) attained acceptable reliability according to both **α** and composite reliability (> = .70, and > =.60, respectively); 2 subscales only attained acceptable values in the upper bound estimate (i.e., composite reliability). Out of the remaining 6, the *Ethics* element had the lowest reliability on its two subscales. We noticed a discrepancy in **α** and composite reliability’s expected behavior (i.e., **α** lower than composite reliability) in the *Motivation* foundation, and *Physical Regulation* foundation subscales.

We found that 42 items (56%) had acceptable individual item reliability (outer loading >.70). Eleven items had unexpected negative loadings - as they were intended to relate positively with their constructs; these were, however, mostly negatively worded items found in *Motivation*, *Physical Regulation* and *Ethics* foundation level subscales.

#### Item analysis (cognitive module)

Table 5 summarizes the preliminary item analysis of the cognitive module of the PPLA-Q. We found a mismatch between intended complexity of the item and its difficulty in 2 of the 5 content groups (i.e., foundational items being answered incorrectly more often that mastery items for the same content); as well as an overall low success in foundational items. Additionally, average difficulty of the items in the module was .50, representing a more difficult test than ideal for maximizing discrimination – .70 to .74, for a test with four, five and six options multiple-choice items [101]. Notwithstanding, 6 items showed good or very good discrimination between lower-knowledge and higher-knowledge students (D > .30). Distractor analysis revealed that 16 (57%) were low functioning distractors (i.e., ≤ 10% of total responses for the item); these were mostly in easier items.

**Table 5:** Difficulty, discrimination, and distractor analysis of items in the Cognitive module (*n* = 40; PPLA-Q version 0.4)

Content			***p***^**1**^	***D***^**2**^	Evaluation^**3**^	Distractor analysis (%)^**4**^					
	Level	Item				Response Option					
						a	b	c	d	e	f
**Nutrition**	Foundation	C1	.95	.08	–	0	**95**	5	0	–	–
	Mastery	C2	.78	.31	+	3	5	13	**80**	–	–
**Fitness and training**	Foundation	C3	.45	.54	++	48	**45**	5	3	–	–
	Mastery	C4	.32	.77	++	**33**	10	15	43	–	–
**Safety and risk**	Foundation	C5*	.40	.54	++	**98**	0	**80**	15	**90**	**45**
	Mastery	C6	.82	.31	+	3	3	**85**	10	–	–
**PA’s Health Benefits**	Foundation	C7	.32	.46	++	**33**	20	15	33	–	–
	Mastery	C8	.80	.23	–	10	8	3	**80**	–	–
**Body Composition**	Foundation	C9	.15	.23	–	**15**	18	53	15	–	–
	Mastery	C10*	.10	.23	–	**43**	13	**90**	5	**53**	–
		**Average p**	**.50**								

* Multiple selection items (“choose all that apply”)

^1^ p - Difficulty index: number of correct responses / total number of responses – higher number means easier item

^2^ D - Discrimination index (generalized ULI): difference in ratio of correct answers in upper and lower third of students

^3^Evaluation cutoffs for discrimination index [100]: >.40 Very good (++); .30–.39 Reasonably good (+); .20–.29 Marginal (−), <.19 Poor (− −)

^4^ Percentage of students choosing option – correct options are bolded

#### Cognitive interviews – 2nd round

Further individual cognitive interviews (*n* = 2) were conducted to probe student’s understating of changes made to the items in the last 3 versions of the PPLA-Q, as well as in items which raised frequent requests for clarification during pilot application. Interviewed students showed good comprehension of the items. Additionally, a minor change was suggested in one of the distractors of the item pertaining to basic safety procedures during PA (C5): substitute “Always drink water” for “Drink water regularly”.

#### Questionnaire refinement

Results of preliminary reliability analysis prompted a detailed analysis of every item and subscale in the psychological and social modules. Based on this, negatively stated items were changed into positively stated ones to improve comprehension, and subsequently, validity and reliability. Minor changes were made to item stems as well, to improve clarity.

Additionally, 11 global assessment items (e.g., “I’m motivated to practice PA”) were introduced into the psychological and social modules to allow for convergent validity assessment through redundancy analysis of the second, third and fourth-order formative constructs [44, 142] in further stages of PPLA-Q development. Of these, 8 targeted each of the elements, 2 targeted the general psychological and social domains, and 1 targeted general PL. Their content followed the respective operational definition stated by the APLF (see Table 1), while adhering to the same structure and rating scale as the remaining items.

Informed by the results of the preliminary item analysis, items in the cognitive module were revised to better conform to the expected difficulty levels (i.e., mastery items harder than foundation ones). We revised low functioning distractors, to make them more plausible to students. C6 was modified from a single selection multiple-choice to *cloze-type* item, with no changes to intended outcome. All revisions in this module were made in consultation with a subject matter expert to ensure technical adequacy and content validity.

Before the next iteration of cognitive interviews, all items were co-validated by non-generating authors to guarantee that clarity was improved, and content validity was left unchanged.

### Version five (v0.5): cognitive interviews

To assess the clarity of items changed between version four (v0.4) and five (v0.5) of the PPLA-Q, 6 students were interviewed. Most Likert-type items were clear and coincident with their intended meaning, except those regarding justice (e.g., “I try to be just”), which led to interpretations related with collaboration and teamwork, instead of the intended meaning regarding ethics/fair-play. In the revised cloze-type item in the cognitive module (C6), one of the students failed to respond to the item according to instructions (i.e., filled in the spaces, instead of circling options that would fill each space), revealing a need to clarify its instructions. According to this data, we fine-tuned all pertaining items. Similarly, informed by the pilot test, we created two different versions of the cognitive module by mirroring the arrangement of options – in the second version, A became D, B became C – hoping to discourage students to share their answers during application and reduce subsequent measurement error.

## Discussion

This article followed the development, content validation, and pilot testing of the first of two instruments that comprise the *Portuguese Physical Literacy Assessment* (PPLA)*: the PPLA-Questionnaire (PPLA-Q)*, it assesses the *psychological*, *social* and part of the *cognitive* domains of PL, inspired by the *Australian Physical Literacy Framework* (APLF) and Portuguese PE syllabus (PPES). Its primary target are high-school students (grade 10 through 12) in PE context. Older adolescents are a critical intervention group – especially in Portugal – given that they possess lower PA levels than their younger peers [33–35]; and will cease to have mandatory and free access to professional guidance in PA and movement, eventually becoming dependent on their PL to participate in meaningful PA, and further advance on their journey.

### Content validity

We gathered evidence on content validity using an iterative process with experts in each subject matter domain (i.e., for each of the modules), and target population. The number of experts per module was considered acceptable and ranged from 4 to 6. Although literature recommends 5–7 experts to rate content validity [47], a minimum of 3 is acceptable for content areas in which expert recruitment might prove difficult [80] – as we argue was the case in this study, given constraints imposed by the COVID-19 pandemic.

PPLA-Q showed evidence for adequate content validity at item level improved throughout multiple revisions. In version 0.2, using **κ**, 96% of the items were rated as good or excellent (>.74) [87, 88] regarding relevance, and 84% considered clear (>.74). Module-wise, a S-CVI/Ave of .90 is considered adequate [89], a cut-off that decreases to .80 for S-CVI/UA, given that it requires universal agreement between all raters [81]. While the psychological module attained an adequate S-CVI on both accounts (.98/.93), the social module did so only on S-CVI/Ave (.90 /.68), and the cognitive failed to achieve both standards (.87/.60); further analysis identified that most items with lower I-CVI in the two latter modules were those generated without a conceptual reference to an existing instrument (i.e., *Culture & Society*). Qualitative suggestions from the experts and advisors augmented quantitative data, targeting concepts in need of rewording or clarification. We then revised and eliminated items to improve content validity across all modules. A targeted revision of the *Culture & Society* scale increased overall social module’s S-CVI to .90/.84 (Ave/UA) on a second round of expert evaluation aimed solely at it (version 0.3).

Multiple rounds of qualitative cognitive interviews were conducted until saturation was achieved (i.e., no new suggestions emerged) [143] with a heterogenous sample of high-school students (*n* = 12), using different versions of the PPLA-Q. These informed improvement on item wording and syntax, to effectively target the intended concepts and reduce ambiguity. During initial stages, students noted lack of clarity in abstract concepts like those from the *Culture & Society* (values, rituals, and traditions of sport/PA), and *Ethics* (justice, honesty, fair play) scales; notwithstanding evidence that iterative revisions clarified these items, further validation efforts should scrutinize their performance. Similarly, despite obtaining evidence for item-level content validity (except for item C2), and subsequent reviews in consultation with a test and assessment expert – based on the qualitative comments of experts and students – we advise further quantitative scrutiny of the cognitive module to establish its module-wise content validity.

### Feasibility

Average completion time for the PPLA-Q was 27 min. Although it might impose a substantial burden upon respondents, diversity of constructs and items used throughout the different modules might have effectively reduced it. Notwithstanding, depuration of subscales in the modules – during the next steps in development – will certainly reduce this time and further improve feasibility.

We had no response errors and low levels of missing data during pilot testing, which might stem from student’s routine exposure to questionnaires using the same item format (i.e., multiple-choice items and Likert-type scales). We also argue that application of the questionnaire during PE class, with the lead investigator present, to clarify any question, might have played a determinant role in this. In one of the application settings (i.e., classroom) it was notorious the student’s urge to copy or share their answers from/with colleagues, especially in the cognitive module. The similarity of this module with usual summative evaluation instruments used in school setting might partially explain this occurrence; non the less, we expect that future use of the two differently arranged versions of the cognitive module (i.e., mirrored distractors) might reduce this.

We experienced a high rate of attrition (≈30%). Constraints imposed by the COVID-19 pandemic might have reduced the number of students completing the questionnaire: both by reducing their willingness to participate, as well as the possibility to be present during application (due to prophylactic lockdown). This number shall inform the sample size calculations in further phases of development, as it is expected that these conditions might endure during next phases.

### Preliminary reliability and item analysis

Results of reliability analysis in the psychological and social modules established preliminary evidence of adequate reliability in 10 out of 16 subscales (**α** > .70 and composite reliability > .60) [97]. Analysis of item reliability highlighted items that were contributing negatively to subscale reliability (outer loading <.70) of the remaining 6 subscales: Upon careful inspection, most of these were negatively worded. Although the use of negative wording might filter out unwarranted responding patterns (e.g. acquiescence), they have the potential to confuse students and compromise validity and reliability [67] by, for example, creating an artificial subconstruct within the intended subscale [144]. As such, these items were altered and then tested for comprehension during subsequent cognitive interviews, with positive results. Further reliability testing is warranted with a bigger sample size, to gather more definite evidence on the reliability of these subscales.

Regarding item analysis of the cognitive module, item difficulty ranged from .10 (very hard) to .95 (very easy), with an average difficulty of .50. Initial evaluation of its 10 items identified 6 good or very good discriminating items (D > .30) [100, 145] (i.e., capable of differentiating knowledge levels among students).

We expected items designed for in the mastery level (i.e., pertinent to deeper learning) to be more difficult than those in the foundation level, within the same content; however, pilot data does not fully support this idea, as foundational items were more difficult than their mastery counterpart in 2 content pairs (C5 & C6, C7 & C8). We identified low-functioning distractors in the mastery level’s C6 & C8 (non-plausible), that increased likelihood of a correct answer, even without full knowledge of the content. Conversely, C5 and C7 (foundation) had characteristics which inflated its difficulty: one of C5’s (multiple selection item about safety during PA) intended “correct” options contained absolute language (“[one should] hydrate during *all the duration* of the activity”), steering respondents away from it; while C7 measured factual knowledge of the recommendations for PA in children and adults, which has been previously shown to be low among adolescents [140] and young adults [136, 141]. A similar phenomenon emerged with C9, which asked respondents to select the Body Mass Index calculation formula – although students might be familiar with the concept they might not recall its formula. Informed by this data, distractors were thoroughly revised.

We would like to acknowledge, that although the methods used here to preliminarily assess the quality of the items followed the *Classical Test Theory* framework, *Item Response Theory* and Rasch models might play a role in further validation efforts, since they expressly integrate the notion of item difficulty (as well, as other possible parameters like discrimination and guessing) into the calculation of student’s scores [43]; this would allow precise student scoring along the learning continuum posited in the development of PPLA. These were not used in this pilot study, given their requirement of larger sample sizes [146].

PPLA as a whole is intended to assess the integrated physical, cognitive, psychological, and social variables that are posited to underpin PL; both to direct the pedagogical action at local, regional and national level in proving a PL-supporting environment, and to inform self-directed changes by the students. Even though it pertains to attitudes, skills and knowledge applied in general PA settings, further adaptation is warranted if it is to be applied to younger students and/or outside of PE. Moreover, we argue that although culture might play a defining role in the representation of PL – as stated by Whitehead [4] – and that the PPLA-Q was designed with this peculiarity in mind, most of its indicators (i.e., items) might be easily adapted to other cultural contexts.

### Strengths and limitations

To our knowledge, this study is the first report of content validity for a measurement instrument of PL designed for grade 10 to 12 adolescents. The content in the tool was inspired by the APLF and the PPES, informed by previous decisions of consortium of experts during a European project (PhyLit). Its development used an iterative process of content validation, using both subject matter experts in each knowledge domain (i.e., cognitive, psychological, and social), as well as target population, resulting in many revisions to improve its clarity and validity.

Although great care was taken to create a heterogeneous sample for the cognitive interviews and pilot test, all participants were nonetheless from a convenience sample from Lisbon’s metropolitan area. Similarly, we could not reach our goal of 6 experts participating in every module. Arguably, the effects of the COVID-19 pandemic might have had an overarching effect on expert availability to participate in the project, and students’ participation rate – through previously discussed constraints. However, we did not collect enough information to extrapolate specific causes for attrition, which could provide additional insights to prepare future studies and further improve feasibility.

Given that only preliminary testing was done regarding reliability and construct validity, further work is warranted and is currently ongoing to establish evidence in this regard, with a statistically adequate sample size.

PPLA inherits the complex nomological network of APLF, as such, some theoretical constructs underwent adjustments in other to be fully integrated into the same model; as such, further robust construct validation needs to ensure adequate dimensionality of each construct chosen, as well as the accuracy, validity, and practical usefulness of the usage of the learning continuum posited through the *foundation* and *mastery* levels. Further studies should also evaluate PPLA-Q’s integration with PPLA-O (in development) to provide a holistic, integrated assessment, as warranted.

Similarly, this effort might allow for depuration of the instrument, contributing to a more parsimonious and shorter version; further improving its feasibility in PE contexts. As the PPLA-Q only targets older adolescents now, future adaptation into earlier age ranges might provide a clearer picture of PL development throughout all school-age.

## Conclusion

This study details the iterative development process of the PPLA-Q as an instrument to assess the psychological, social, and part of the cognitive domain of PL in grade 10 to 12 adolescents (15–18 years). It also provides evidence for adequate content validity at item level, and, except for the cognitive module, at module level. It was improved through multiple rounds of expert and target-population consultation. This instrument has also shown good feasibility within PE settings, and gathered preliminary evidence in favor of its reliability for application in older adolescents. Further validation efforts are needed to reinforce these conclusions, establish evidence of construct validity, and study PPLA-Q’s integration with the PPLA-O (an instrument in development to assess the remaining domains of PL) within the PPLA framework to provide feedback to support older adolescents in their PL journey.

## Supplementary Information

**Additional file 1.** Expert information. Description: Information about experts that participated in the content validation of the PPLA-Q.

**Additional file 2.** Descriptive Content Validity Results. Description: Results of Content Validity Index and coefficient Kappa calculations.

**Additional file 3.** Portuguese Physical Literacy Assessment – Questionnaire (version 0.6). Description: Version 0.6 of the Portuguese Physical Literacy Assessment Questionnaire, both in English and Portuguese (original language).

## Data Availability

Supporting data is not available as participants of this study did not explicitly agree to share their data publicly. The latest development version of the questionnaire used - *Portuguese Physical Literacy Assessment Questionnaire* (version 0.6) - is available in Additional file 3, both in its original Portuguese version, and translated to English for reader’s convenience; interested readers might procure an updated version with the lead author.

## References

[1] Sport Australia (2019). Australian physical literacy framework.

[2] Whitehead M (2001). The concept of physical literacy. Eur J Phys Educ.

[3] Whitehead M (2007). Physical literacy: philosophical considerations in relation to developing a sense of self, universality and propositional knowledge. Sport Ethics Philos.

[4] Physical literacy: throughout the lifecourse. Routledge; 2010.

[5] Telama R (2009). Tracking of physical activity from childhood to adulthood: a review. Obes Facts.

[6] Telama R (2014). Tracking of physical activity from early childhood through youth into adulthood. Med Sci Sports Exerc.

[7] Guthold R, Stevens GA, Riley LM, Bull FC (2018). Worldwide trends in insufficient physical activity from 2001 to 2016: a pooled analysis of 358 population-based surveys with 1·9 million participants. Lancet Glob Health.

[8] Guthold R, Stevens GA, Riley LM, Bull FC (2020). Global trends in insufficient physical activity among adolescents: a pooled analysis of 298 population-based surveys with 1·6 million participants. Lancet Child Adolesc Health.

[9] UNESCO. Quality Physical Education (QPE): guidelines for policy makers. Paris: UNESCO Publishing; 2015.

[10] Onofre M. A Qualidade da Educação Física como Essência da Promoção de uma Cidadania Ativa e Saudável. Retos: nuevas tendencias en educación física, deporte y recreación. 2017;31:328–33.

[11] Woods C, Moyna N, Quinlan A (2010). The children’s sport participation and physical activity study (CSPPA study).

[12] Corbin CB (2016). Implications of physical literacy for research and practice: a commentary. Res Q Exerc Sport.

[13] Dudley D (2015). A conceptual model of observed physical literacy. Phys Educ.

[14] Dudley D, Cairney J, Wainwright N, Kriellaars D, Mitchell D (2017). Critical considerations for physical literacy policy in public health, recreation, sport, and education agencies. Quest.

[15] Whitehead M (2013). Definition of physical literacy and clarification of related issues. ICSSPE J Sport Sci Phys Educ.

[16] Whitehead M (2013). The history and development of physical literacy. ICSSPE J Sport Sci Phys Educ.

[17] Edwards L, Bryant A, Keegan R, Morgan K, Jones A (2017). Definitions, foundations and associations of physical literacy: a systematic review. Sports Med.

[18] Liu Y, Chen S (2021). Physical literacy in children and adolescents: definitions, assessments, and interventions. Eur Phys Educ Rev.

[19] Martins J, et al. International approaches to the definition, philosophical tenets, and core elements of physical literacy: a scoping review. PROSPECTS. 2020. 10.1007/s11125-020-09466-1.

[20] Robinson DB, Randall L, Barrett J (2018). Physical literacy (Mis)understandings: what do leading physical education teachers know about physical literacy?. J Teach Phys Educ.

[21] Pot N, Whitehead ME, Durden-Myers EJ (2018). Physical literacy from philosophy to practice. J Teach Phys Educ.

[22] Young L, O’Connor J, Alfrey L (2019). Physical literacy: a concept analysis. Sport Educ Soc.

[23] Biggs J, Collis K. Evaluating the Quality of Learning: The SOLO Taxonomy (Structure of Observed Learning Outcomes). New York: Academic Press; 1982.

[24] Keegan R, Barnett L, Dudley D (2017). Literature sampling to inform development of a physical literacy definition and standard for Australia.

[25] Keegan R (2019). Defining physical literacy for application in Australia: a modified Delphi method. J Teach Phys Educ.

[26] Edwards L (2017). ‘Measuring’ physical literacy and related constructs: a systematic review of empirical findings. Sports Med.

[27] Shearer C (2021). Assessments related to the physical, affective and cognitive domains of physical literacy amongst children aged 7–11.9 years: A Systematic Review. Sports Med Open.

[28] Francis CE (2016). The Canadian assessment of physical literacy: development of a model of Children’s capacity for a healthy, active lifestyle through a Delphi process. J Phys Act Health.

[29] Gunnell KE, Longmuir PE, Barnes JD, Belanger K, Tremblay MS. Refining the Canadian Assessment of Physical Literacy based on theory and factor analyses. BMC Public Health. 2018;18(Suppl 2):131–45. 10.1186/s12889-018-5899-2 PMC6167769 30285682

[30] Cairney J (2018). A construct validation study of PLAYfun. Med Sci Sports Exerc.

[31] Longmuir PE, Gunnell KE, Barnes JD, Belanger K, Leduc G, Woodruff SJ, et al. Canadian Assessment of Physical Literacy Second Edition: a streamlined assessment of the capacity for physical activity among children 8 to 12 years of age. BMC Public Health. 2018;18(Suppl 2):169–80. 10.1186/s12889-018-5902-y PMC6167760 30285687

[32] Blanchard J, Van Wyk N, Ertel E, Alpous A, Longmuir PE. Canadian assessment of physical literacy in grades 7-9 (12-16 years): preliminary validity and descriptive results. J Sports Sci. 10.1080/02640414.2019.1689076. 10.1080/02640414.2019.1689076 31703541

[33] Baptista F (2012). Prevalence of the Portuguese population attaining sufficient physical activity. Med Sci Sports Exerc.

[34] Martins J (2019). Trends and age-related changes of physical activity among Portuguese adolescent girls from 2002–2014: highlights from the health behavior in school-aged children study. J Phys Act Health.

[35] Matos MG, Equipa Aventura Social (2018). A Saúde dos Adolescentes Portugueses após a Recessão - Dados nacionais 2018.

[36] Crum B (1993). Conventional thought and practice in physical education: problems of teaching and implications for change. Quest.

[37] Tinning R (2015). ‘I don’t read fiction’: academic discourse and the relationship between health and physical education. Sport Educ Soc.

[38] Durden-Myers EJ, Green NR, Whitehead ME. Implications for promoting physical literacy progress. J Teach Phys Educ. 2018;37. 10.1123/jtpe.2018-0131.

[39] Harlen W. Assessment of Learning. London: SAGE Publications Ltd; 2007.

[40] Onofre M, Costa J, Martins J, Quitério, Ana. Physical Education and School Sport in Portugal. In: Naul R, Scheuer C, editors. Research on Physical Education and School Sport in Europe. Aachen: Meyer & Meyer; 2020.

[41] Taxonomy of education objectives: the classification of education goals: handbook 2 - affective domain. David McKay; 1964.

[42] Cairney J, Clark H, Dudley D, Kriellaars D (2019). Physical literacy in children and youth—a construct validation study. J Teach Phys Educ.

[43] Andrich D, Marais IA (2019). Course in Rasch measurement theory: measuring in the educational, social and health sciences.

[44] Hair JF, Hult G, Ringle C, Sarstedt M. A primer on partial least squares structural equation modeling (PLS-SEM). Second edition. Los Angeles: Sage; 2017.

[45] Jarvis CB, MacKenzie SB, Podsakoff PM (2003). A critical review of construct indicators and measurement model misspecification in marketing and consumer research. J Consum Res.

[46] Armstrong TS, Cohen MZ, Eriksen L, Cleeland C (2005). Content validity of self-report measurement instruments: an illustration from the development of the brain tumor module of the M.D. Anderson symptom inventory. Oncol Nurs Forum.

[47] Boateng GO, Neilands TB, Frongillo EA, Melgar-Quiñonez HR, Young SL. Best practices for developing and validating scales for health, social, and behavioral research: a primer. Front Public Health. 2018;6(149):18. 10.3389/fpubh.2018.00149 PMC6004510 29942800

[48] Longmuir PE, Woodruff SJ, Boyer C, Lloyd M, Tremblay MS. Physical Literacy Knowledge Questionnaire: feasibility, validity, and reliability for Canadian children aged 8 to 12 years. BMC Public Health. 2018;18(Suppl 2):19–29. 10.1186/s12889-018-5890-y PMC6167766 30285679

[49] Dudley D, Keegan R, Barnett L (2017). Physical Literacy: Informing a Definition and Standard for Australia.

[50] Ministério da Educação (2001). Programa Nacional Educação Física : Ensino Secundário.

[51] Ministério da Educação (2001). Programa Nacional Educação Física (Reajustamento) : Ensino Básico 3^o^Ciclo.

[52] Ministério da Educação (2018). Aprendizagens Essenciais: Educação Física.

[53] Deci EL, Ryan RM (2000). The ‘what’ and ‘why’ of goal pursuits: human needs and the self-determination of behavior. Psychol Inq.

[54] Deci EL, Ryan RM (2008). Self-determination theory: a macrotheory of human motivation, development, and health. Can Psychol Can.

[55] Markland D, Tobin V (2004). A modification to the Behavioural regulation in exercise questionnaire to include an assessment of amotivation. J Sport Exerc Psychol.

[56] Wilson PM, Rogers WT, Rodgers WM, Wild TC (2006). The psychological need satisfaction in exercise scale. J Sport Exerc Psychol.

[57] Deci EL, Ryan RM, editors. Handbook of Self-Determination Research. Rochester, New York: University of Rochester Press; 2002.

[58] Goleman D. Emotional Intelligence: Why It Can Matter More Than IQ. New York: Bantam; 2005.

[59] Wong C-S, Law KS (2002). The effects of leader and follower emotional intelligence on performance and attitude: an exploratory study. Leadersh Q.

[60] Siedentop D (1998). What is sport education and how does it work?. J Phys Educ Recreat Dance.

[61] Gibbs JC. Moral development and reality: beyond the theories of Kohlberg, Hoffman, and Haidt. Third edition. Oxford: Oxford University Press; 2014.

[62] Kohlberg L. Development of Moral Character and Moral Ideology. In: Hoffman LW, Hoffman ML, editors. Review of Child Development Research: Volume 1. New York: Russell Sage Foundation; 1964.

[63] Hassandra M, Goudas M, Hatzigeorgiadis A. Development of a questionnaire assessing fair play in elementary school physical education. Athlitki Psychol. 2002:105–26.

[64] Hellison D. Teaching Personal and Social Responsibility Through Physical Activity. 3rd edition. Champaign: Human Kinetics; 2011.

[65] Li W, Wright PM, Rukavina PB, Pickering M (2008). Measuring students’ perceptions of personal and social responsibility and the relationship to intrinsic motivation in urban physical education. J Teach Phys Educ.

[66] World Health Organization. Global recommendations on physical activity for health. Geneva: World Health Organization; 2010. 26180873

[67] DeVellis R. Scale Development: Theory and Applications. Los Angeles: SAGE Publications Ltd; 2017.

[68] Price LR. Psychometric Methods Theory into Practice. New York: The Guilford Press; 2017.

[69] Clark L, Watson D (1995). Constructing validity: basic issues in objective scale development. Psychol Assess.

[70] Bollen KA. Structural equations with latent variables. New York: Wiley; 1989.

[71] Kyriazos TA, Stalikas A (2018). Applied psychometrics: the steps of scale development and standardization process. Psychology.

[72] Artino AR, La Rochelle JS, Dezee KJ, Gehlbach H (2014). Developing questionnaires for educational research: AMEE Guide No. 87. Med Teach.

[73] DeCastellarnau A (2018). A classification of response scale characteristics that affect data quality: a literature review. Qual Quant.

[74] Furr RM. Scale Construction and Psychometrics for Social and Personality Psychology. London: SAGE Publications Ltd; 2011.

[75] Considine J, Botti M, Thomas S (2005). Design, format, validity and reliability of multiple choice questions for use in nursing research and education. Collegian.

[76] Scully D (2017). Constructing Multiple-Choice Items to Measure Higher-Order Thinking.

[77] Polit DF, Beck CT, Owen SV (2007). Is the CVI an acceptable indicator of content validity? Appraisal and recommendations. Res Nurs Health.

[78] Willis GB, Artino AR (2013). What do our respondents think We’re asking? Using cognitive interviewing to improve medical education surveys. J Grad Med Educ.

[79] Wynd CA, Schmidt B, Schaefer MA (2003). Two quantitative approaches for estimating content validity. West J Nurs Res.

[80] Lynn MR (1986). Determination and quantification of content validity. Nurs Res.

[81] Davis LL (1992). Instrument review: getting the most from a panel of experts. Appl Nurs Res.

[82] Zamanzadeh V (2015). Design and Implementation Content Validity Study: Development of an instrument for measuring Patient-Centered Communication.

[83] RStudio Team. RStudio: Integrated Development for R. Boston: RStudio, PBC; 2020.

[84] R Core Team. R: A language and environment for statistical compution. Vienna, Austria: R Foundation for Statistical Computing; 2020.

[85] Polit DF, Beck CT (2006). The content validity index: are you sure you know what’s being reported? Critique and recommendations. Res Nurs Health.

[86] Cohen J (1960). A coefficient of agreement for nominal scales. Educ Psychol Meas.

[87] Fleiss J (1971). Measuring nominal scale agreement among many raters. Psychol Bull.

[88] Cicchetti DV, Sparrow SA (1981). Developing criteria for establishing interrater reliability of specific items: applications to assessment of adaptive behavior. Am J Ment Defic.

[89] Waltz CF, Strickland O, Lenz ER. Measurement in nursing and health research. New York: Springer; 2010.

[90] American Educational Research Association, American Psychological Association, National Council on Measurement in Education. Standards for Educational and Psychological Testing. Washington, D.C: American Educational Research Association; 2014.

[91] Hertzog MA (2008). Considerations in determining sample size for pilot studies. Res Nurs Health.

[92] Johanson GA, Brooks GP (2010). Initial scale development: sample size for pilot studies. Educ Psychol Meas.

[93] Hair JF, Risher JJ, Sarstedt M, Ringle CM (2019). When to use and how to report the results of PLS-SEM. Eur Bus Rev.

[94] Ringle CM, Wende S, Becker J-M. SmartPLS 3: SmartPLS; 2015.

[95] Hair Jr. JF, Sarstedt M, Ringle CM, Gudergan S. Advanced Issues in Partial Least Squares Structural Equation Modelling. Los Angeles: SAGE Publications; 2018.

[96] Kline P. The Handbook of Psychological Testing. Abingdon, Oxfordshire: Routledge; 2000.

[97] Nunnaly J, Bernstein I. Psychometric Theory. New York: McGraw-Hill; 1994.

[98] Willse J (2018). Classical Test Theory Functions (CTT).

[99] Martinková P, Drabinová A (2019). ShinyItemAnalysis for teaching psychometrics and to enforce routine analysis of educational tests. R J.

[100] Ebel R, Frisbie D. Essentials of Educational Measurement. Englewood Cliffs, New Jersey: Prentice-Hall, Inc; 1991.

[101] Lord F. The relation of the reliability of multiple-choice tests to the distribution of item dificulties. Psychometrika. 1952;17.

[102] Towns MH (2014). Guide to developing high-quality, reliable, and valid multiple-choice assessments. J Chem Educ.

[103] Teixeira PJ, Carraça EV, Markland D, Silva MN, Ryan RM. Exercise, physical activity, and self-determination theory: A systematic review. 2012;9(78):30. 10.1186/1479-5868-9-78. 10.1186/1479-5868-9-78 PMC3441783 22726453

[104] Ryan RM, Deci EL. Overview of self-determination theory: An organismic-dialectical perspective. In: Handbook of self-determination research. Rochester: University of Rochester Press; 2002. p. 3–33.

[105] Cortis C (2017). Psychological determinants of physical activity across the life course: a ‘DEterminants of DIet and physical ACtivity’ (DEDIPAC) umbrella systematic literature review. PLoS One.

[106] Vasconcellos D, Parker P, Hilland T, Cinelli R, Owen K, Kapsal N, et al. Self-determination theory applied to physical education: A systematic review and meta-analysis. J Educ Psychol. 2019;112:1444–69.

[107] Gagné M (2010). The motivation at work scale: validation evidence in two languages. Educ Psychol Meas.

[108] Babic MJ (2014). Physical activity and physical self-concept in youth: systematic review and Meta-analysis. Sports Med.

[109] Bandura A. Social foundations of thought and action: A social cognitive theory. Englewood Cliffs, New Jersey: Prentice-Hall, Inc; 1986.

[110] Sweet SN, Fortier MS, Strachan SM, Blanchard CM (2012). Testing and integrating self-determination theory and self-efficacy theory in a physical activity context. Can Psychol Can.

[111] Hughes A, Galbraith D, White D (2011). Perceived competence: a common Core for self-efficacy and self-concept?. J Pers Assess.

[112] Bandura A. Self-Efficacy: The Exercise of Control. New York: W.H. Freeman and Company; 1997.

[113] Baumeister RF, Vohs KD (2007). Self-regulation, Ego depletion, and motivation: motivation and Ego depletion. Soc Personal Psychol Compass.

[114] Zeidner M, Matthews G, Roberts R (2012). What we know about emotional intelligence: how it affects learning, work, relationships, and our mental health.

[115] Ubago-Jiménez JL, González-Valero G, Puertas-Molero P, García-Martínez I. Development of emotional intelligence through physical activity and sport practice. A Systematic Review. Behav Sci. 2019;9(4):44. 10.3390/bs9040044. 10.3390/bs9040044 PMC6523064 31022863

[116] Nielson WR, Jensen MP, Karsdorp PA, Vlaeyen JWS (2013). Activity pacing in chronic pain: concepts, evidence, and future directions. Clin J Pain.

[117] Lazarus RS, Folkman S. Stress, Appraisal, and Coping. New York: Springer Publishing Company; 1984.

[118] Pons J, Viladrich C, Ramis Y, Polman R. The mediating role of coping between competitive anxiety and sport commitment in adolescent athletes. Span J Psychol. 2018;21. 10.1017/sjp.2018.8 29576037

[119] Earley PC, Ang S. Cultural Intelligence: Individual Interactions Across Cultures. Stanford: Stanford University Press; 2003.

[120] Mazurkiewicz M (2011). Some observations about ritual in sport. Stud Phys Cult Tour.

[121] Vallerand RJ (2006). Passion in sport: a look at determinants and affective experiences. J Sport Exerc Psychol.

[122] Eastman ST, Riggs KE (1994). Televised sports and ritual: fan experiences. Sociol Sport J.

[123] Wallhead TL, Garn AC, Vidoni C (2013). Sport education and social goals in physical education: relationships with enjoyment, relatedness, and leisure-time physical activity. Phys Educ Sport Pedagogy.

[124] Brooks AW, Schroeder J, Risen JL, Gino F, Galinsky AD, Norton MI, et al. Don’t stop believing: Rituals improve performance by decreasing anxiety. Organ Behav Hum Decis Process. 2016;137:71–85.

[125] Bronikowska M (2019). Fair play in physical education and beyond. Sustainability.

[126] Simon RL, Torres CR, Hager PF. Fair Play: The Ethics of Sport. 4th Edition. Boulder, Colorado: Westview Press; 2015.

[127] Turiel E. Morality and Prosocial Judgments and Behavior. In: Schroeder DA, Graziano WG, editors. The Oxford Handbook of Prosocial Behavior. Oxford: Oxford University Press; 2015.

[128] Hassandra M, Goudas M, Hatzigeorgiadis A (2003). Attitudes towards fair play in physical education: the role of intrinsic motivation and gender. Proceedings, XIth European Congress of Sport Psychology.

[129] Hassandra M, Goudas M, Hatzigeorgiadis A, Theodorakis Y (2007). A fair play intervention program in school Olympic education. Eur J Psychol Educ.

[130] Pozo P, Grao-Cruces A, Pérez-Ordás R (2018). Teaching personal and social responsibility model-based programmes in physical education: a systematic review. Eur Phys Educ Rev.

[131] Taylor IM, Ntoumanis N, Standage M, Spray CM (2010). Motivational predictors of physical education students’ effort, exercise intentions, and leisure-time physical activity: a multilevel linear growth analysis. J Sport Exerc Psychol.

[132] Cox A, Duncheon N, McDavid L (2009). Peers and teachers as sources of relatedness perceptions, motivation, and affective responses in physical education. Res Q Exerc Sport.

[133] Martins J, Marques A, Sarmento H, Carreiro da Costa F (2015). Adolescents’ perspectives on the barriers and facilitators of physical activity: a systematic review of qualitative studies. Health Educ Res.

[134] Leptokaridou ET, Vlachopoulos SP, Papaioannou AG (2015). Associations of autonomy, competence, and relatedness with enjoyment and effort in elementary school physical education: the mediating role of self-determined motivation. Hell J Psychol.

[135] Abula K, Gröpel P, Chen K, Beckmann J (2018). Does knowledge of physical activity recommendations increase physical activity among Chinese college students? Empirical investigations based on the transtheoretical model. J Sport Health Sci.

[136] Haase A, Steptoe A, Sallis JF, Wardle J (2004). Leisure-time physical activity in university students from 23 countries: associations with health beliefs, risk awareness, and national economic development. Prev Med.

[137] Vaara JP, Vasankari T, Koski HJ, Kyröläinen H. Awareness and knowledge of physical activity recommendations in Young adult men. Front Public Health. 2019;7:310. 10.3389/fpubh.2019.00310. 10.3389/fpubh.2019.00310 PMC6831523 31737590

[138] Fredriksson SV, et al. How are different levels of knowledge about physical activity associated with physical activity behaviour in Australian adults? PLoS One. 2018;13(11):e0207003. 10.1371/journal.pone.0207003. 10.1371/journal.pone.0207003 PMC6261553 30485310

[139] Xu F, et al. Awareness of knowledge and practice regarding physical activity: a population-based prospective, observational study among students in Nanjing, China. PLoS One. 2017;12(6):e0179518. 10.1371/journal.pone.0179518. 10.1371/journal.pone.0179518 PMC5473587 28622354

[140] Marques A, Martins J, Sarmento H, Rocha L, da Costa FC (2015). Do students know the physical activity recommendations for health promotion?. J Phys Act Health.

[141] Martins J (2019). Physical activity recommendations for health: knowledge and perceptions among college students. Retos Nuevas Tend En Educ Física Deporte Recreación.

[142] Cheah J-H, Sarstedt M, Ringle CM, Ramayah T, Ting H (2018). Convergent validity assessment of formatively measured constructs in PLS-SEM: on using single-item versus multi-item measures in redundancy analyses. Int J Contemp Hosp Manag.

[143] Rodrigues IB, Adachi JD, Beattie KA, MacDermid JC (2017). Development and validation of a new tool to measure the facilitators, barriers and preferences to exercise in people with osteoporosis. BMC Musculoskelet Disord.

[144] van Sonderen E, Sanderman R, Coyne JC (2013). Ineffectiveness of reverse wording of questionnaire items: Let’s learn from cows in the rain. PLoS One.

[145] Lord FM (1952). The relation of the reliability of multiple-choice tests to the distribution of item difficulties. Psychometrika.

[146] Haladyna TM. Developing and validating multiple-choice test items. Mahwah, New Jersey: Lawrence Erlbaum Associates; 2004.

