# Supplementary material for: Portuguese Physical Literacy Assessment Questionnaire (PPLA-Q) for adolescents (15–18 years) from grades 10–12: development, content validation and pilot testing
Source: BMC Public Health. 2021 Nov 29;21:2183. doi: 10.1186/s12889-021-12230-5 (PMC8628133; doi:10.1186/s12889-021-12230-5)
Supplement: Supplementary file 1 — Additional file 1. Expert information. Description: Information about experts that participated in the content validation of the PPLA-Q. [file 12889_2021_12230_MOESM1_ESM.docx]

**Additional file 1**

**Supplementary Table S1. Information about experts that participated in the content validation of the PPLA-Q**

| **Expert** | **PPLA-Q Domain** | **Expertise** | **#Citations ^1^** | **h-index^1^** | **Round participation** |
| --- | --- | --- | --- | --- | --- |
| 1 | Cognitive | Test development, educational assessment | 201 | NA | 1^st^ |
| 2 | Cognitive | Health promotion and public Health | 3468 | 28 | 1^st^ |
| 3 | Cognitive | Curriculum development, PE didactics | 80 | NA | 1^st^ |
| 4 | Cognitive | Curriculum development, PE didactics, developer of the PPES | NA | NA | 1^st^ |
| 5 | Psychological | Sport psychology, decision-making in sport | 16699 | 72 | 1^st^ |
| 6 | Psychological | Scale development and validation, sport psychology | 1052 | 17 | 1^st^* |
| 7 | Psychological | Sport psychology, behavioral change | 3025 | 25 | 1^st^ |
| 8 | Social | Sport sociology, school ethnography | 59 | 5 | 1^st^ & 2^nd^ |
| 9 | Social and Psychological | Sport psychology and pedagogy, scale validation | 5287 | 35 | 1^st^ |
| 10 | All | Healthy and active lifestyles, PE Didactics | 1162 | 18 | 1^st^ & 2^nd^ |
| 11 | All | PE didactics, Teacher education | 3014 | 27 | 1^st^ |
| 12 | All | PE didactics, Teacher education | 1167 | 16 | 2^nd^ |
| *Note*. All experts were professors at Graduate-level Education. NA – Not available; PE – Physical Education; PPES – Portuguese Physical Education syllabus.  *Qualitative evaluation only.  ^1^Citation data obtained from each expert’s Google Scholar profile in January 2021. | | | | | |
