# Supplementary material for: Portuguese Physical Literacy Assessment Questionnaire (PPLA-Q) for adolescents (15–18 years) from grades 10–12: development, content validation and pilot testing
Source: BMC Public Health. 2021 Nov 29;21:2183. doi: 10.1186/s12889-021-12230-5 (PMC8628133; doi:10.1186/s12889-021-12230-5)
Supplement: Supplementary file 2 — Additional file 2. Descriptive Content Validity Results. Description: Results of Content Validity Index and coefficient Kappa calculations. [file 12889_2021_12230_MOESM2_ESM.docx]

**Additional file 2**

**Supplementary Table S2. Item CVI, kappa coefficient and evaluation of each item (PPLA-Q version 0.2)**

| Item | **Relevance** | | | | |  | | **Clarity** | | | | |
| --- | --- | --- | --- | --- | --- | --- | --- | --- | --- | --- | --- | --- |
|  | Number of Experts | Experts Giving Rating of 3 or 4 | I-CVI | **κ** *^1^* | Evaluation*^2^* |  | Number of Experts | | Experts Giving Rating of 3 or 4 | Proportion of Agreement | **κ** *^1^* | Evaluation*^3^* |
| **Cognitive Module** | | |  |  |  |  |  | |  |  |  |  |
| C1 | 6 | 6 | 1 | 1 | Excellent |  | 6 | | 6 | 1 | 1 | Clear |
| C2 | 6 | 2 | .33 | .13 | Eliminate |  | 6 | | 3 | .50 | .27 | Review |
| C3 | 5 | 5 | 1 | 1 | Excellent |  | 6 | | 6 | 1 | 1 | Clear |
| C4 | 5 | 5 | 1 | 1 | Excellent |  | 6 | | 5 | .83 | .82 | Clear |
| C5 | 5 | 5 | 1 | 1 | Excellent |  | 6 | | 6 | 1 | 1 | Clear |
| C6 | 5 | 4 | .80 | .76 | Excellent |  | 6 | | 4 | .67 | .56 | Review |
| C7 | 5 | 5 | 1 | 1 | Excellent |  | 6 | | 5 | .83 | .82 | Clear |
| C8 | 5 | 4 | .80 | .76 | Excellent |  | 6 | | 2 | .33 | .13 | Review |
| C9 | 5 | 5 | 1 | 1 | Excellent |  | 6 | | 5 | .83 | .82 | Clear |
| C10 | 5 | 4 | .80 | .76 | Excellent |  | 6 | | 6 | 1 | 1 | Clear |
| **S-CVI/Ave** |  |  | .87 |  |  |  |  | |  |  |  |  |
| **S-CVI/UA** |  |  | .60 |  |  |  |  | |  |  |  |  |
| **Psychological Module** | | |  |  |  |  |  | |  |  |  |  |
| P1 | 5 | 5 | 1 | 1 | Excellent |  | 5 | | 5 | 1 | 1 | Clear |
| P2 | 5 | 5 | 1 | 1 | Excellent |  | 5 | | 5 | 1 | 1 | Clear |
| P3 | 5 | 5 | 1 | 1 | Excellent |  | 5 | | 5 | 1 | 1 | Clear |
| P4 | 5 | 5 | 1 | 1 | Excellent |  | 5 | | 5 | 1 | 1 | Clear |
| P5 | 5 | 5 | 1 | 1 | Excellent |  | 5 | | 5 | 1 | 1 | Clear |
| P6 | 5 | 5 | 1 | 1 | Excellent |  | 5 | | 5 | 1 | 1 | Clear |
| P7 | 5 | 5 | 1 | 1 | Excellent |  | 5 | | 5 | 1 | 1 | Clear |
| P8 | 5 | 5 | 1 | 1 | Excellent |  | 5 | | 4 | .80 | .76 | Clear |
| P9 | 5 | 5 | 1 | 1 | Excellent |  | 5 | | 4 | .80 | .76 | Clear |
| P10 | 5 | 5 | 1 | 1 | Excellent |  | 5 | | 5 | 1 | 1 | Clear |
| P11 | 5 | 5 | 1 | 1 | Excellent |  | 5 | | 5 | 1 | 1 | Clear |
| P12 | 5 | 5 | 1 | 1 | Excellent |  | 5 | | 5 | 1 | 1 | Clear |
| P13 | 5 | 5 | 1 | 1 | Excellent |  | 5 | | 5 | 1 | 1 | Clear |
| P14 | 5 | 5 | 1 | 1 | Excellent |  | 5 | | 5 | 1 | 1 | Clear |
| P15 | 5 | 5 | 1 | 1 | Excellent |  | 5 | | 5 | 1 | 1 | Clear |
| P16 | 5 | 4 | .80 | .76 | Excellent |  | 5 | | 4 | .80 | .76 | Clear |
| P17 | 5 | 5 | 1 | 1 | Excellent |  | 5 | | 5 | 1 | 1 | Clear |
| P18 | 5 | 5 | 1 | 1 | Excellent |  | 5 | | 5 | 1 | 1 | Clear |
| P19 | 5 | 5 | 1 | 1 | Excellent |  | 5 | | 5 | 1 | 1 | Clear |
| P20 | 5 | 5 | 1 | 1 | Excellent |  | 5 | | 5 | 1 | 1 | Clear |
| P21 | 5 | 5 | 1 | 1 | Excellent |  | 5 | | 5 | 1 | 1 | Clear |
| P22 | 5 | 5 | 1 | 1 | Excellent |  | 5 | | 5 | 1 | 1 | Clear |
| P23 | 5 | 5 | 1 | 1 | Excellent |  | 5 | | 5 | 1 | 1 | Clear |
| P24 | 5 | 4 | .80 | .76 | Excellent |  | 5 | | 5 | 1 | 1 | Clear |
| P25 | 5 | 5 | 1 | 1 | Excellent |  | 5 | | 4 | .80 | .76 | Clear |
| P26 | 5 | 5 | 1 | 1 | Excellent |  | 5 | | 5 | 1 | 1 | Clear |
| P27 | 5 | 5 | 1 | 1 | Excellent |  | 5 | | 5 | 1 | 1 | Clear |
| P28 | 5 | 5 | 1 | 1 | Excellent |  | 5 | | 5 | 1 | 1 | Clear |
| P29 | 5 | 5 | 1 | 1 | Excellent |  | 5 | | 5 | 1 | 1 | Clear |
| P30 | 5 | 5 | 1 | 1 | Excellent |  | 5 | | 5 | 1 | 1 | Clear |
| P31 | 5 | 5 | 1 | 1 | Excellent |  | 5 | | 5 | 1 | 1 | Clear |
| P32 | 5 | 5 | 1 | 1 | Excellent |  | 5 | | 5 | 1 | 1 | Clear |
| P33 | 5 | 5 | 1 | 1 | Excellent |  | 5 | | 5 | 1 | 1 | Clear |
| P34 | 5 | 5 | 1 | 1 | Excellent |  | 5 | | 5 | 1 | 1 | Clear |
| P35 | 4 | 4 | 1 | 1 | Excellent |  | 4 | | 4 | 1 | 1 | Clear |
| P36 | 5 | 4 | .80 | .76 | Excellent |  | 5 | | 5 | 1 | 1 | Clear |
| P37 | 4 | 4 | 1 | 1 | Excellent |  | 4 | | 3 | .75 | .67 | Review |
| P38 | 4 | 4 | 1 | 1 | Excellent |  | 3 | | 3 | 1 | 1 | Clear |
| P39 | 4 | 4 | 1 | 1 | Excellent |  | 3 | | 3 | 1 | 1 | Clear |
| P40 | 4 | 4 | 1 | 1 | Excellent |  | 4 | | 2 | .50 | .20 | Review |
| **S-CVI/Ave** |  |  | .98 |  |  |  |  | |  |  |  |  |
| **S-CVI/UA** |  |  | .93 |  |  |  |  | |  |  |  |  |
| **Social Module** | | |  |  |  |  |  | |  |  |  |  |
| S1 | 4 | 3 | .75 | .67 | Good |  | 3 | | 1 | .33 | -.07 | Review |
| S2 | 4 | 3 | .75 | .67 | Good |  | 4 | | 4 | 1 | 1 | Clear |
| S3 | 4 | 2 | .50 | .20 | Eliminate |  | 4 | | 3 | .75 | .67 | Review |
| S4 | 4 | 4 | 1 | 1 | Excellent |  | 4 | | 3 | .75 | .67 | Review |
| S5 | 4 | 4 | 1 | 1 | Excellent |  | 4 | | 4 | 1 | 1 | Clear |
| S6 | 4 | 3 | .75 | .67 | Good |  | 3 | | 3 | 1 | 1 | Clear |
| S7 | 4 | 4 | 1 | 1 | Excellent |  | 3 | | 3 | 1 | 1 | Clear |
| S8 | 4 | 3 | .75 | .67 | Good |  | 3 | | 3 | 1 | 1 | Clear |
| S9 | 4 | 4 | 1 | 1 | Excellent |  | 3 | | 3 | 1 | 1 | Clear |
| S10 | 4 | 4 | 1 | 1 | Excellent |  | 4 | | 4 | 1 | 1 | Clear |
| S11 | 4 | 4 | 1 | 1 | Excellent |  | 4 | | 4 | 1 | 1 | Clear |
| S12 | 4 | 4 | 1 | 1 | Excellent |  | 4 | | 4 | 1 | 1 | Clear |
| S13 | 4 | 4 | 1 | 1 | Excellent |  | 4 | | 4 | 1 | 1 | Clear |
| S14 | 4 | 4 | 1 | 1 | Excellent |  | 3 | | 3 | 1 | 1 | Clear |
| S15 | 4 | 4 | 1 | 1 | Excellent |  | 4 | | 4 | 1 | 1 | Clear |
| S16 | 4 | 4 | 1 | 1 | Excellent |  | 4 | | 4 | 1 | 1 | Clear |
| S17 | 4 | 4 | 1 | 1 | Excellent |  | 4 | | 4 | 1 | 1 | Clear |
| S18 | 4 | 4 | 1 | 1 | Excellent |  | 4 | | 4 | 1 | 1 | Clear |
| S19 | 4 | 4 | 1 | 1 | Excellent |  | 4 | | 4 | 1 | 1 | Clear |
| S20 | 4 | 3 | .75 | .67 | Good |  | 4 | | 4 | 1 | 1 | Clear |
| S21 | 4 | 3 | .75 | .67 | Good |  | 4 | | 3 | .75 | .67 | Review |
| S22 | 4 | 3 | .75 | .67 | Good |  | 4 | | 3 | .75 | .67 | Review |
| S23 | 4 | 3 | .75 | .67 | Good |  | 4 | | 4 | 1 | 1 | Clear |
| S24 | 3 | 3 | 1 | 1 | Excellent |  | 4 | | 4 | 1 | 1 | Clear |
| S25 | 3 | 3 | 1 | 1 | Excellent |  | 4 | | 4 | 1 | 1 | Clear |
| S26 | 3 | 3 | 1 | 1 | Excellent |  | 4 | | 4 | 1 | 1 | Clear |
| S27 | 3 | 3 | 1 | 1 | Excellent |  | 4 | | 4 | 1 | 1 | Clear |
| S28 | 4 | 4 | 1 | 1 | Excellent |  | 4 | | 4 | 1 | 1 | Clear |
| S29 | 4 | 3 | .75 | .67 | Good |  | 4 | | 3 | .75 | .67 | Review |
| S30 | 4 | 2 | .50 | .20 | Eliminate |  | 4 | | 4 | 1 | 1 | Clear |
| S31 | 4 | 3 | .75 | .67 | Good |  | 4 | | 3 | .75 | .67 | Review |
| S32 | 4 | 4 | 1 | 1 | Excellent |  | 4 | | 4 | 1 | 1 | Clear |
| S33 | 4 | 4 | 1 | 1 | Excellent |  | 4 | | 4 | 1 | 1 | Clear |
| S34 | 4 | 2 | .50 | .20 | Eliminate |  | 4 | | 3 | .75 | .67 | Review |
| S35 | 4 | 4 | 1 | 1 | Excellent |  | 4 | | 4 | 1 | 1 | Clear |
| S36 | 4 | 4 | 1 | 1 | Excellent |  | 4 | | 4 | 1 | 1 | Clear |
| S37 | 4 | 4 | 1 | 1 | Excellent |  | 3 | | 2 | .67 | .47 | Review |
| S38 | 4 | 4 | 1 | 1 | Excellent |  | 3 | | 3 | 1 | 1 | Clear |
| S39 | 4 | 4 | 1 | 1 | Excellent |  | 3 | | 3 | 1 | 1 | Clear |
| S40 | 4 | 4 | 1 | 1 | Excellent |  | 3 | | 3 | 1 | 1 | Clear |
| **S-CVI/Ave** |  |  | .90 |  |  |  |  | |  |  |  |  |
| **S-CVI/UA** |  |  | .68 |  |  |  |  | |  |  |  |  |
| I-CVI- Item Content Validity Index; Ave- Average; UA- Universal Agreement; κ – kappa coefficient; S-CVI- Scale Content Validity Index.  *^1^*Multirater modified kappa designating agreement on relevance: κ= (I-CVI - pc)/(1 -pc), with pc (probability of a chance occurrence) computed using the formula for a binomial random variable, with one specific outcome described in Polit et al. (2007)  *^2^*Evaluation criteria for kappa, using guidelines described in Cicchetti and Sparrow (1981) and Fleiss (1981): Fair kappa of .40 to .59; Good kappa .60 to .74; and Excellent kappa > .74.  *^3^*Modified criteria for kappa: Needs Revision < .74; Clear > .74 | | | | | | | | | | | | |

**Supplementary Table S3. Social Module’s Item CVI, kappa coefficient and evaluation (PPLA-Q version 0.3)**

| Item | **Relevance** | | | | |  | | **Clarity** | | | | |
| --- | --- | --- | --- | --- | --- | --- | --- | --- | --- | --- | --- | --- |
|  | Number of Experts | Number Giving Rating of 3 or 4 | I-CVI | **κ** *^1^* | Evaluation*^2^* |  | Number of Experts | | Experts Giving Rating of 3 or 4 | Proportion of Agreement | **κ** *^1^* | Evaluation*^3^* |
| S1* | 3 | 3 | 1 | 1 | Excellent |  | 3 | | 3 | 1 | 1 | Clear |
| S2* | 3 | 3 | 1 | 1 | Excellent |  | 3 | | 3 | 1 | 1 | Clear |
| S3* | 3 | 3 | 1 | 1 | Excellent |  | 3 | | 2 | .67 | .47 | Review |
| S4* | 3 | 3 | 1 | 1 | Excellent |  | 3 | | 2 | .67 | .47 | Review |
| S5* | 3 | 3 | 1 | 1 | Excellent |  | 3 | | 2 | .67 | .47 | Review |
| S6* | 3 | 3 | 1 | 1 | Excellent |  | 3 | | 3 | 1 | 1 | Clear |
| S7* | 3 | 3 | 1 | 1 | Excellent |  | 3 | | 2 | .67 | .47 | Review |
| S8* | 3 | 3 | 1 | 1 | Excellent |  | 3 | | 3 | 1 | 1 | Clear |
| S9* | 3 | 3 | 1 | 1 | Excellent |  | 3 | | 1 | .33 | −.07 | Review |
| S10 | 4 | 4 | 1 | 1 | Excellent |  | 4 | | 4 | 1 | 1 | Clear |
| S11 | 4 | 4 | 1 | 1 | Excellent |  | 4 | | 4 | 1 | 1 | Clear |
| S12 | 4 | 4 | 1 | 1 | Excellent |  | 4 | | 4 | 1 | 1 | Clear |
| S13 | 4 | 4 | 1 | 1 | Excellent |  | 4 | | 4 | 1 | 1 | Clear |
| S14 | 4 | 4 | 1 | 1 | Excellent |  | 3 | | 3 | 1 | 1 | Clear |
| S15 | 4 | 4 | 1 | 1 | Excellent |  | 4 | | 4 | 1 | 1 | Clear |
| S16 | 4 | 4 | 1 | 1 | Excellent |  | 4 | | 4 | 1 | 1 | Clear |
| S17 | 4 | 4 | 1 | 1 | Excellent |  | 4 | | 4 | 1 | 1 | Clear |
| S18 | 4 | 4 | 1 | 1 | Excellent |  | 4 | | 4 | 1 | 1 | Clear |
| S19 | 4 | 4 | 1 | 1 | Excellent |  | 4 | | 4 | 1 | 1 | Clear |
| S20 | 4 | 3 | .75 | .67 | Good |  | 4 | | 4 | 1 | 1 | Clear |
| S21 | 4 | 3 | .75 | .67 | Good |  | 4 | | 3 | .75 | .67 | Review |
| S22 | 4 | 3 | .75 | .67 | Good |  | 4 | | 3 | .75 | .67 | Review |
| S23 | 4 | 3 | .75 | .67 | Good |  | 4 | | 4 | 1 | 1 | Clear |
| S24 | 3 | 3 | 1 | 1 | Excellent |  | 4 | | 4 | 1 | 1 | Clear |
| S25 | 3 | 3 | 1 | 1 | Excellent |  | 4 | | 4 | 1 | 1 | Clear |
| S26 | 3 | 3 | 1 | 1 | Excellent |  | 4 | | 4 | 1 | 1 | Clear |
| S27 | 3 | 3 | 1 | 1 | Excellent |  | 4 | | 4 | 1 | 1 | Clear |
| S28 | 4 | 4 | 1 | 1 | Excellent |  | 4 | | 4 | 1 | 1 | Clear |
| S29 | 4 | 3 | .75 | .67 | Good |  | 4 | | 3 | .75 | .67 | Review |
| S30 | 4 | 3 | .75 | .67 | Good |  | 4 | | 3 | .75 | .67 | Review |
| S31 | 4 | 4 | 1 | 1 | Excellent |  | 4 | | 4 | 1 | 1 | Clear |
| S32 | 4 | 4 | 1 | 1 | Excellent |  | 4 | | 4 | 1 | 1 | Clear |
| S33 | 4 | 4 | 1 | 1 | Excellent |  | 4 | | 4 | 1 | 1 | Clear |
| S34 | 4 | 4 | 1 | 1 | Excellent |  | 4 | | 4 | 1 | 1 | Clear |
| S35* | 3 | 3 | 1 | 1 | Excellent |  | 3 | | 3 | 1 | 1 | Clear |
| S36 | 4 | 4 | 1 | 1 | Excellent |  | 3 | | 3 | 1 | 1 | Clear |
| S37 | 4 | 4 | 1 | 1 | Excellent |  | 3 | | 3 | 1 | 1 | Clear |
| S38 | 4 | 4 | 1 | 1 | Excellent |  | 3 | | 3 | 1 | 1 | Clear |
| S-CVI/Ave |  |  | .96 |  |  |  |  | |  |  |  |  |
| S-CVI/UA |  |  | .84 |  |  |  |  | |  |  |  |  |
| I-CVI- Item Content Validity Index; Ave- Average; UA- Universal Agreement; κ – kappa coefficient; S-CVI- Scale Content Validity Index.  *Items included in 2^nd^ round of expert validation.  *^1^*Multirater modified kappa designating agreement on relevance: κ=(I-CVI - pc)/(1 -pc), with pc (probability of a chance occurrence) computed using the formula for a binomial random variable, with one specific outcome described in Polit et al. (2007)  *^2^*Evaluation criteria for kappa, using guidelines described in Cicchetti and Sparrow (1981) and Fleiss (1981): Fair kappa of .40 to .59; Good kappa .60 to .74; and Excellent kappa > .74  *^3^*Modified criteria for kappa: Needs Revision < .74; Clear > .74 | | | | | | | | | | | | |
