# Supplementary material for: Portuguese Physical Literacy Assessment Questionnaire (PPLA-Q) for adolescents (15–18 years) from grades 10–12: development, content validation and pilot testing
Source: BMC Public Health. 2021 Nov 29;21:2183. doi: 10.1186/s12889-021-12230-5 (PMC8628133; doi:10.1186/s12889-021-12230-5)
Supplement: Supplementary file 3 — Additional file 3. Portuguese Physical Literacy Assessment – Questionnaire (version 0.6). Description: Version 0.6 of the Portuguese Physical Literacy Assessment Questionnaire, both in English and Portuguese (original language). [file 12889_2021_12230_MOESM3_ESM.pdf]

Additional file 3 - PPLA-Q v0.6

PPLA-Q version 0.6 – original (Portuguese) and English translation

English version

For the next question group, you should remember that PA = Physical Activity(ies), and includes every situation that require movement, like your Physical Education classes, sport-based activities (team or individual), rhythmic activities (e.g., dance), exercise (e.g., strength training, jogging), and any activities that you use as a mean of transportation or in your spare time.

In each statement below, select how much it describes you, circling one of the options from 0 to 4 0 = ***Not at all***; 1= ***Slightly***; 2 =***Moderately***; 3 =***Quite a lot***; 4 = ***Totally****)*.

How much do these statements describe you?	Not al all	Slightly	Moderately	Quite a lot	Totally
P1. I am motivated to practice PA	0	1	2	3	4
P2. I practice PA because others tell me I should	0	1	2	3	4
P3. I feel guilty when I do not practice PA	0	1	2	3	4
P4. I feel bad about myself when I do not practice PA	0	1	2	3	4
P5. I feel pressured by others to practice PA	0	1	2	3	4
P6. I practice PA because I feel others would be unhappy if I did not	0	1	2	3	4
P7. I practice PA because it is fun	0	1	2	3	4
P8. I feel good when I practice PA	0	1	2	3	4
P9. I consider PA a part of me	0	1	2	3	4
P10. I value the benefits of PA	0	1	2	3	4
P11. I see PA as a fundamental part of who I am	0	1	2	3	4
P12. I enjoy practicing PA	0	1	2	3	4
P13. I feel confident to practice PA	0	1	2	3	4
**In Physical Activity contexts:**					
P14. I am confident in my abilities	0	1	2	3	4
P15. I can participate with success	0	1	2	3	4
P16. I consider myself competent	0	1	2	3	4
P17. I trust my skills	0	1	2	3	4
P18. I feel good about the way I am able to participate	0	1	2	3	4
P19. I can participate in PA that I consider challenging	0	1	2	3	4

This is a development version of the Portuguese Physical Literacy Assessment Questionnaire. For the most updated version, please contact the lead author.

P20. I know how to become more confident in myself	0	1	2	3	4
P21. I feel competent even when I am criticized	0	1	2	3	4
P22. I believe in myself even when I lose	0	1	2	3	4
P23. I can manage my emotions	0	1	2	3	4
P24. I can recognize other's emotions	0	1	2	3	4
P25. I can recognize my emotions	0	1	2	3	4
**In Physical Activity contexts:**					
P26. I am sensitive to the feelings of others	0	1	2	3	4
P27. I understand what others feel	0	1	2	3	4
P28. I can identify what I feel	0	1	2	3	4
P29. I can anticipate what I will feel	0	1	2	3	4
P30. I can deal with difficulties rationally	0	1	2	3	4
P31. I can manage my emotions when necessary	0	1	2	3	4
P32. I have a good control of my emotions	0	1	2	3	4
P33. I can manage my effort	0	1	2	3	4
P34. I know when I am tired	0	1	2	3	4
P35. I can recognize changes in my breathing	0	1	2	3	4
P36. I can recognize changes in my heart rate	0	1	2	3	4
P37. I recognize my physical limits	0	1	2	3	4
P38. I can recognize the effect that different intensities have in me	0	1	2	3	4
P39. I use strategies to manage my effort	0	1	2	3	4
P40. I can anticipate when I will be fatigued	0	1	2	3	4
P41. I can control my fatigue	0	1	2	3	4
P42. I take action to improve my physical skills	0	1	2	3	4
**In the different contexts of my life:**					
P43. I feel more motivated to reach my goals because I practice PA	0	1	2	3	4
P44. I feel more confident in my skills because I practice PA	0	1	2	3	4
P45. I am better at controlling my emotions because I practice PA	0	1	2	3	4
P46. I am better at controlling my fatigue because I practice PA	0	1	2	3	4

How much do these statements describe you?	Not al all	Slightly	Moderately	Quite a lot	Totally
S1. I believe that the cultural aspects of PA are important (e.g., its rituals, terminology, clothing, values)	0	1	2	3	4
S2. I participate in PA rituals (e.g., greetings, hymns/chants, cheers, applauses)	0	1	2	3	4
S3. I use specific PA terminology (e.g., names of technics and tactics, names of equipment, idioms)	0	1	2	3	4
S4. I use specific clothing of the PA I am practicing	0	1	2	3	4
S5. I watch PA events (e.g., competitions, spectacles, shows)	0	1	2	3	4
S6. I like to keep up with PA events (e.g., competitions, spectacles, shows)]	0	1	2	3	4
S7. I am interested in the cultural aspects of PA (e.g., its rituals, terminology, clothing, values)]	0	1	2	3	4
S8. I encourage others to watch PA events (e.g., competitions, spectacles, shows)	0	1	2	3	4
S9. I encourage others to participate in each PA's culture (e.g., rituals, terminology, clothing)]	0	1	2	3	4
S10. I can lead a healthy and active life	0	1	2	3	4
**In Physical Activity contexts:**					
S11. I work well with others	0	1	2	3	4
S12. I try to behave correctly and justly	0	1	2	3	4
S13. I respect my adversaries	0	1	2	3	4
S14. I follow the rules	0	1	2	3	4
S15.I cheat if it brings me benefits	0	1	2	3	4
S16. I respect the decisions of authorities (e.g., referee, umpire, coach/teacher)]	0	1	2	3	4
S17. I behave according to fair-play / sport ethics 'principles	0	1	2	3	4
S18. I understand the importance of fair play/ sport ethics' principles	0	1	2	3	4
S19. I take action to make others behave according to fair play/sport ethic	0	1	2	3	4
S20. I follow the rules, even if unsupervised	0	1	2	3	4
S21. I behave according to fair play/sport ethics' principles on my initiative	0	1	2	3	4

This is a development version of the Portuguese Physical Literacy Assessment Questionnaire. For the most updated version, please contact the lead author.

S22. I take action for others to follow the rules	0	1	2	3	4
S23. I collaborate with others	0	1	2	3	4
S24. I am sympathetic with others	0	1	2	3	4
S25. I control my behavior towards others	0	1	2	3	4
S26. I respect others	0	1	2	3	4
S27. I cooperate with others	0	1	2	3	4
S28. I encourage others	0	1	2	3	4
S29. I care about others' success	0	1	2	3	4
S30. I help others achieve success	0	1	2	3	4
S31. I am helpful with others	0	1	2	3	4
S32. I have a positive relationship with others	0	1	2	3	4
S33. I interact with others	0	1	2	3	4
S34. I share a common goal with others	0	1	2	3	4
S35. I feel close to others	0	1	2	3	4
S36. I feel a sense of camaraderie with others	0	1	2	3	4
S37. I take action to improve my relationship with others	0	1	2	3	4
S38. I know how to improve my relationship with others	0	1	2	3	4
S39. I care about my relationship with others	0	1	2	3	4
**In the different contexts of my life:**					
S40. I am more involved in other cultural activities (e.g., theater, music) because I practice PA	0	1	2	3	4
S41. I am more honest and just because I practice PA	0	1	2	3	4
S42. I collaborate more with others because I practice PA	0	1	2	3	4
S43. I have better relationships with others because I practice PA	0	1	2	3	4

This question group will be about your opinion about topics of physical activity and healthy lifestyles.
Read each question attentively and circle your option(s).

C1. Select the option which represents a healthy and balanced meal.

(A) Chicken soup, cheeseburger, apple, soda.
(B) Vegetable soup, grilled salmon with sweet potatoes and baked vegetables, pear, water.
(C) Garlic bread, carbonara pasta with salad, pear, water.
(D) Bean soup, fried shrimp patties with tomato rice, apple, soda.

C2. In a normal day, Maria total calorie intake is 2000 kcal, and her total caloric expenditure is 2200 kcal.

Select the option which describes Maria's energetical balance for that day and its effects on her bodyweight.

(A) She will have a negative energetic balance and will lose weight.
(B) She will have a positive energetic balance and will gain weight.
(C) She will have a positive energetic balance and will lose weight.
(D) She will have a negative energetic balance and will lose weight.

C3. The "capacity to capture and use oxygen to produce energy" and the "capacity to move one or more joints through their complete range of motion" describe, respectively:

(A) muscular strength, and flexibility.
(B) aerobic fitness, and flexibility.
(C) aerobic fitness, and muscular strength.
(D) speed, and muscular strength.

C4. João has low strength and flexibility and wants to improve those capacities.

Select the option which describes adequate training methods for João.

	Training	Frequency	Volume per muscle group/joint
(A)	**Strength**	**2- 3 non consecutive sessions per week**	**1 to 3 sets of 12 repetions**
	**Flexibility**	**5-7 sessions per week**	**30 seconds per exercise**
(B)	Strength	1 session per week	1 to 3 sets of 12 repetions
	Flexibility	5-7 sessions per week	30 seconds per exercise
(C)	Strength	2- 3 consecutive sessions per week	1 to 3 sets of 12 repetions
	Flexibility	5-7 sessions per week	30 seconds per exercise
(D)	Strength	2- 3 non consecutive sessions per week	1 to 3 sets of 5 repetions
	Flexibility	5-7 sessions per week	30 seconds per exercise

C5. Select **all** the options which describe basic actions to avoid injuries during physical activity.

(A) Perform a warmup in the beginning of the activity, and a cool down at the end of the activity.
(B) In the event of an injury, apply something hot immediately.
(C) Use adequate protective equipment.
(D) Have a meal 10 minutes before an activity.
(E) Follow the safety rules of the activity.
(F) Drink water regularly during the activity.

C6. Fill in the text by selecting in the following table the adequate option for each space.

A competitive athlete is thinking about using anabolic steroids. This practice is __1.__ since it __2.__ fair-play, 3.__ sport performance, and __4.__ health.

1.	2.	3.	4.
**(A)** safe<br>**(B) forbidden**<br>**(C)** permitted	**(A)** increases<br>**(B) decreases**<br>**(C)** has no effect on	**(A) increases**<br>**(B)** decreases<br>**(C)** has no effect on	**(A) increases the risk of negative consequences for**<br>**(B)** decreases the risk of negative consequences for<br>**(C)** has no effect on

C7. The World Health Organization has defined recommendations for Physical Activity.

Select the option which describes the general guidelines for children and adolescents, and for adults.

(A) Children and adolescents: 60 minutes per day with moderate to vigorous intensity; adults: 150 minutes per week with moderate to vigorous intensity.
(B) Children and adolescents: 150 minutes per week with moderate to vigorous intensity; **adults**: 150 minutes per week with moderate to vigorous intensity
(C) Children and adolescents: 30 minutes per day with moderate to vigorous intensity; **adults**: 150 minutes per week with moderate to vigorous intensity.
(D) Children and adolescents: 60 minutes per day with moderate to vigorous intensity; **adults**: 90 minutes per week with moderate to vigorous intensity.

C8. Select the option that correctly links each type of physical training with its impact on health.

Types of Fitness Training
1.Strength training
2.Flexibility training
3. Aerobic fitness training

Impacts on health
A. Better blood pressure and cardiovascular health
B. Increased resting heart rate
C. Increased mobility and ability to perform day-to-day activities
D. Increased coordination difficulties
E. Better bone, tendon, and ligament health, and reduced injury risk

(A) 1-D, 2-C, 3-A
(B) 1-A, 2-B, 3-C
(C) 1-E, 2-B, 3-D
(D) 1-E, 2-C, 3-A

C9. The calculation formula for the Body Mass Index (BMI) is

(A) $\frac{\boldsymbol{Weight\ (kg)}}{\boldsymbol{Height\ (m)^2}}$

(B) $\frac{Height\ (cm)^2}{Weight\ (kg)}$

(C) $\frac{Weight\ (kg)}{Height\ (cm)^2}$

(D) $\frac{Height\ (m)}{Weight\ (kg)^2}$

C10. Based on the analysis of the two tables provided, select **all** true statements.

Table 1

Individual	Sex	Age	Body Mass Index	Physical Activity
1	Male	15	18	Occasional
2	Female	16	26,3	Occasional
3	Male	20	27	Regular

Table 2. Reference values for BMI (FITschool)

	Body Mass Index – Healthy Range			
	Girls		Boys	
Age	>	<	>	<
15	16,0	23,8	16,3	23,1
16	16,3	24,3	16,7	23,9
17	16,4	24,6	17,2	24,6
18+	18,5	25,0	18,5	25,0

(A) Individual 1 should practice physical activity regularly.
(B) Individual 1 is below the healthy range of BMI.
(C) Individual 2 should reduce her weight.
(D) Individual 3 should maintain his weight.
(E) Individual 2 has higher risk for disease than individual 3.

Check that you answered all questions.

Thank you for your invaluable participation!

Additional file 3 - PPLA-Q v0.6

Versão Portuguesa

Para responderes ao próximo grupo, deves relembrar que **AF = Atividade(s) Física(s)**, e que inclui todas as situações que impliquem movimento, como por exemplo, as aulas de Educação Física, as atividades desportivas (coletivas ou individuais), as atividades rítmicas (ex. dança), o exercício físico (ex. treino de força, *jogging*), e as atividades físicas que faças para te deslocar ou nos teus tempos livres.

Para cada afirmação abaixo deves selecionar o quanto essa te descreve circundando uma das opções de 0 a 4 (**0= Nada, 1= Pouco, 2= Moderadamente, 3= Bastante, 4= Totalmente**).

**Como é que estas frases te descrevem?**	Nada	Pouco	Moderadamente	Bastante	Totalmente
P1. Sinto-me motivado(a) para praticar AF	0	1	2	3	4
P2. Faço AF porque outras pessoas dizem que devo fazer	0	1	2	3	4
P3. Sinto-me culpado(a) quando não faço AF	0	1	2	3	4
P4. Sinto-me mal comigo mesmo(a) quando não faço AF	0	1	2	3	4
P5. Sinto-me pressionado(a) pelos outros para fazer AF	0	1	2	3	4
P6. Faço AF porque outras pessoas vão ficar insatisfeitas comigo se não fizer	0	1	2	3	4
P7. Faço AF porque é divertido	0	1	2	3	4
P8. Sinto-me bem quando faço AF	0	1	2	3	4
P9. Considero que a AF faz parte de mim	0	1	2	3	4
P10. Valorizo os benefícios de praticar AF	0	1	2	3	4
P11. Vejo a AF como parte fundamental de quem sou	0	1	2	3	4
P12. Gosto de praticar AF	0	1	2	3	4
P13. Sinto-me confiante para praticar AF	0	1	2	3	4
**Em situações de Atividade Física:**					
P14. Sinto-me confiante nas minhas habilidades	0	1	2	3	4
P15. Consigo participar com sucesso	0	1	2	3	4
P16. Considero-me bom/boa praticante	0	1	2	3	4
P17. Confio nas minhas capacidades	0	1	2	3	4
P18. Sinto-me bem com a forma como consigo participar	0	1	2	3	4
P19. Consigo participar em AF que considero desafiantes	0	1	2	3	4
P20. Sei como fazer para me tornar mais confiante	0	1	2	3	4
P21. Sinto-me capaz mesmo quando sou criticado(a)	0	1	2	3	4

This is a development version of the Portuguese Physical Literacy Assessment Questionnaire. For the most updated version, please contact the lead author.

P22. Acredito na minha capacidade mesmo quando falho	0	1	2	3	4
P23. Consigo gerir as minhas emoções	0	1	2	3	4
P24. Reconheço as emoções dos outros	0	1	2	3	4
P25. Consigo reconhecer as minhas emoções	0	1	2	3	4
**Em situações de Atividade Física:**					
P26. Sou um(a) bom(a) observador(a) das emoções dos outros	0	1	2	3	4
P27. Percebo o que os outros sentem	0	1	2	3	4
P28. Consigo identificar o que sinto	0	1	2	3	4
P29. Consigo antecipar as minhas emoções	0	1	2	3	4
P30. Consigo lidar com as dificuldades racionalmente	0	1	2	3	4
P31. Consigo gerir as minhas emoções quando necessário	0	1	2	3	4
P32. Tenho um bom controlo das minhas emoções	0	1	2	3	4
P33. Consigo gerir o meu esforço	0	1	2	3	4
P34. Sei quando me sinto cansado(a)	0	1	2	3	4
P35. Consigo aperceber-me de mudanças na minha respiração	0	1	2	3	4
P36. Consigo reconhecer mudanças no meu ritmo cardíaco	0	1	2	3	4
P37. Tenho noção dos meus limites físicos	0	1	2	3	4
P38. Reconheço o efeito que diferentes intensidades de esforço têm em mim	0	1	2	3	4
P39. Utilizo estratégias para gerir o meu esforço	0	1	2	3	4
P40. Consigo antecipar quando irei ficar cansado(a)	0	1	2	3	4
P41. Consigo controlar o meu cansaço	0	1	2	3	4
P42. Faço por melhorar as minhas capacidades físicas	0	1	2	3	4
**Nas diversas situações da minha vida:**					
P43. Sinto-me mais motivado(a) a atingir os meus objetivos porque pratico AF	0	1	2	3	4
P 44. Sinto-me mais confiante nas minhas capacidades porque pratico AF	0	1	2	3	4
P45. Controlo melhor as minhas emoções porque pratico AF	0	1	2	3	4
P46. Controlo melhor o meu cansaço porque pratico AF	0	1	2	3	4

Como é que estas frases te descrevem?	Nada	Pouco	Moderadamente	Bastante	Totalmente
S1. Acredito que os aspetos culturais da(s) AF são importantes (ex. os seus rituais, termos específicos, roupa, valores)	0	1	2	3	4
S2. Participo em rituais das AF (ex. saudações, hinos/cânticos, gritos, aplausos)	0	1	2	3	4
S3. Utilizo termos específicos das AF (ex. nomes de técnicas ou táticas, nomes de equipamento, expressões)	0	1	2	3	4
S4. Utilizo vestuário específico da AF que estou a praticar	0	1	2	3	4
S5. Assisto a eventos de AF (ex. competições, demonstrações, programas)	0	1	2	3	4
S6. Gosto de acompanhar eventos de AF (ex. competições, demonstrações, programas)	0	1	2	3	4
S7. Interesso-me pelos aspetos culturais das AF (ex. os seus rituais, termos específicos, roupa, valores)	0	1	2	3	4
S8. Incentivo os outros a assistirem a eventos de AF (ex. competições, demonstrações, programas)	0	1	2	3	4
S9. Encorajo os outros a participar na cultura de cada AF (ex. rituais, termos específicos, roupa)	0	1	2	3	4
S10. Consigo ter uma vida ativa e saudável	0	1	2	3	4
**Em situações de Atividade Física:**					
S11. Trabalho bem com os outros	0	1	2	3	4
S12. Tento ter comportamentos corretos e justos	0	1	2	3	4
S13. Respeito os meus adversários	0	1	2	3	4
S14. Cumpro as regras	0	1	2	3	4
S15. Faço batota, se isso me trouxer benefícios	0	1	2	3	4
S16. Respeito as decisões de autoridades (ex. árbitro, juiz, treinador/professor)	0	1	2	3	4
S17. Cumpro os princípios do *fair-play*/ética desportiva	0	1	2	3	4
S18. Compreendo a importância dos princípios do *fair-play*/ética desportiva	0	1	2	3	4
S19. Faço para que os outros respeitem o *fair-play*/ética desportiva	0	1	2	3	4
S20. Cumpro as regras, mesmo sem supervisão	0	1	2	3	4
S21. Cumpro os princípios do *fair-play*/ética desportiva por iniciativa própria	0	1	2	3	4

This is a development version of the Portuguese Physical Literacy Assessment Questionnaire. For the most updated version, please contact the lead author.

S22. Faço para que os outros cumpram as regras	0	1	2	3	4
S23. Colaboro com os outros	0	1	2	3	4
S24. Sou compreensivo(a) com os outros	0	1	2	3	4
S25. Controlo o meu comportamento para com os outros	0	1	2	3	4
S26. Respeito os outros	0	1	2	3	4
S27. Coopero com os outros	0	1	2	3	4
S28. Encorajo os outros	0	1	2	3	4
S29. Preocupo-me com o sucesso dos outros	0	1	2	3	4
S30. Ajudo os outros a terem sucesso	0	1	2	3	4
S31. Sou prestável com os outros	0	1	2	3	4
S32. Tenho um relacionamento positivo com os outros	0	1	2	3	4
S33. Interajo com os outros	0	1	2	3	4
S34. Partilho um objetivo comum com os outros	0	1	2	3	4
S35. Sinto-me próximo(a) dos outros	0	1	2	3	4
S36. Sinto que há camaradagem entre mim e os outros	0	1	2	3	4
S37. Faço por melhorar o meu relacionamento com os outros	0	1	2	3	4
**Em situações de Atividade Física:**					
S38. Sei como fazer para melhorar o meu relacionamento com os outros	0	1	2	3	4
S39. Preocupo-me com a qualidade do relacionamento que tenho com os outros	0	1	2	3	4
**Nas diversas situações da minha vida:**					
S40. Envolvo-me mais noutras atividades culturais (ex. teatro, música) porque pratico AF	0	1	2	3	4
S41. Sou mais correto(a) e justo(a) porque pratico AF	0	1	2	3	4
S42. Colaboro mais com os outros porque pratico AF	0	1	2	3	4
S43. Tenho melhores relações com os outros porque pratico AF	0	1	2	3	4

Additional file 3 - PPLA-Q v0.6

Este grupo de questões procura conhecer a tua opinião em relação a temas relacionados com a prática de Atividade Física e estilos de vida saudáveis. **Lê cada uma com atenção e coloca um círculo em volta da(s) alínea(s) escolhida(s).**

C1. Seleciona a opção que representa uma refeição saudável e equilibrada.

(A) Canja de galinha, cheeseburger, maçã, refrigerante.
(B) Sopa de legumes, salmão grelhado com batata-doce e vegetais assados, pera, água.
(C) Pão de alho, massa à carbonara com salada, pera, água.
(D) Sopa de feijão, rissóis de camarão fritos com arroz de tomate, maçã, refrigerante.

C2. Num dia normal, a Maria ingere no total 2000 kcal e tem um dispêndio calórico total de 2200 kcal.

Seleciona a opção que descreve o balanço energético da Maria nesse dia e as consequências no seu peso.

(A) Ela terá um balanço energético negativo e ganhará peso.
(B) Ela terá um balanço energético positivo e ganhará peso.
(C) Ela terá um balanço energético positivo e perderá peso.
(D) Ela terá um balanço energético negativo e perderá peso.

C3. A "capacidade de captar e utilizar o oxigénio para produzir energia" e a "capacidade de mover uma ou mais articulações através da sua total amplitude" descrevem, respetivamente

(A) a força muscular e a flexibilidade.
(B) a aptidão aeróbia e a flexibilidade.
(C) a aptidão aeróbia e a força muscular.
(D) a velocidade e a força muscular.

C4. O João tem pouca força e flexibilidade e quer melhorar estas capacidades.

Seleciona a opção que descreve métodos de treino adequados para o João.

	Treino	Frequência	Volume por grupo muscular/articulação
**(A)**	**Força**	**2- 3 vezes não consecutivas por semana**	**1 a 3 séries de 12 repetições**
	**Flexibilidade**	**5-7 vezes por semana**	**30 segundos por alongamento**
**(B)**	Força	1 vez por semana	1 a 3 séries de 12 repetições
	Flexibilidade	5-7 vezes por semana	30 segundos por alongamento
**(C)**	Força	2- 3 vezes consecutivas por semana	1 a 3 séries de 12 repetições
	Flexibilidade	1 vez por semana	30 segundos por alongamento
**(D)**	Força	2- 3 vezes não consecutivas por semana	1 a 3 séries de 5 repetições
	Flexibilidade	5-7 vezes por semana	30 segundos por alongamento

This is a development version of the Portuguese Physical Literacy Assessment Questionnaire. For the most updated version, please contact the lead author.

C5. Seleciona **todas** as opções que descrevem procedimentos básicos para prevenir lesões e danos físicos durante a prática de atividade física.

(A) Aquecer no início da atividade e retornar à calma no final.
(B) Aplicar quente imediatamente, em caso de lesão.
(C) Utilizar material de proteção adequado.
(D) Comer uma refeição 10 minutos antes da atividade.
(E) Cumprir as regras de segurança da modalidade.
(F) Hidratar regularmente enquanto se estiver em atividade.

6. Completa o texto, selecionando na tabela a opção adequada a **cada espaço**.

Uma atleta de competição está a ponderar utilizar esteroides anabolizantes. Esta prática é __1.__ dado que _2.__ verdade desportiva, _3.__ desempenho desportivo e __4.__ saúde.

1.	2.	3.	4.
**(A)** segura<br>**(B) proibida**<br>**(C)** permitida	**(A)** aumenta a<br>**(B) diminui a**<br>**(C)** não tem efeito na	**(A) aumenta o**<br>**(B)** diminui o<br>**(C)** não tem efeito no	**(A) aumenta a probabilidade de problemas de**<br>**(B)** diminui a probabilidade de problemas de<br>**(C)** não tem efeito na

C7. A Organização Mundial de Saúde definiu recomendações para a prática de Atividade Física.

Seleciona a opção que descreve as recomendações gerais para crianças e adolescentes, e para adultos.

(A) Crianças e adolescentes: 60 minutos por dia com intensidade moderada a vigorosa; adultos: 150 minutos por semana com intensidade moderada a vigorosa.
(B) Crianças e adolescentes: 150 minutos por semana com intensidade moderada a vigorosa; **adultos**: 150 minutos por semana com intensidade moderada a vigorosa
(C) Crianças e adolescentes: 30 minutos por dia com intensidade moderada a vigorosa; **adultos**: 150 minutos por semana com intensidade moderada a vigorosa.
(D) Crianças e adolescentes: 60 minutos por dia com intensidade moderada a vigorosa; **adultos**: 90 minutos por semana com intensidade moderada a vigorosa.

C8. Seleciona a opção que associa corretamente o tipo de treino físico aos seus impactos na saúde.

Tipos de Treino Físico	Impactos na Saúde
1.Treino de Força	A. Melhoria da tensão arterial e da saúde cardiovascular.
	B. Aumento da frequência cardíaca de repouso
2.Treino de Flexibilidade	C. Maior mobilidade e capacidade para realizar atividades do dia-a-dia
	D. Maiores dificuldades de coordenação
3.Treino de Aptidão Aeróbia	E. Melhoria nos ossos, tendões e ligamentos e menor risco de lesões

(A) 1-D, 2-C, 3-A
(B) 1-A, 2-B, 3-C
(C) 1-E, 2-B, 3-D
(D) 1-E, 2-C, 3-A

C9. A fórmula de cálculo do Índice de Massa Corporal (IMC) é

(A) $\frac{\boldsymbol{Peso\ (kg)}}{\boldsymbol{Altura\ (m)^2}}$

(B) $\frac{Altura\ (cm)^2}{Peso\ (kg)}$

(C) $\frac{Peso\ (kg)}{Altura\ (cm)^2}$

(D) $\frac{Altura\ (m)}{Peso\ (kg)^2}$

C10. Seleciona, com base na análise das duas tabelas, **todas** as afirmações verdadeiras.

Tabela 1.

Indivíduo	Sexo	Idade	Índice Massa Corporal	Prática Atividade Física
1	Masculino	15	18	Irregular
2	Feminino	16	26,3	Irregular
3	Masculino	20	27	Regular

Tabela 2. Valores de referência de IMC (FITescola)

	Índice De Massa Corporal			
	Zona Saudável			
	Raparigas		Rapazes	
Idade	>	<	>	<
15	16,0	23,8	16,3	23,1
16	16,3	24,3	16,7	23,9
17	16,4	24,6	17,2	24,6
18+	18,5	25,0	18,5	25,0

(A) O indivíduo 1 deveria praticar atividade física regularmente.
(B) O indivíduo 1 encontra-se abaixo da zona saudável de IMC.
(C) O indivíduo 2 deveria reduzir o seu peso.
(D) O indivíduo 3 deveria manter o seu peso.
(E) O indivíduo 2 tem maior risco de doenças que o indivíduo 3.

Confirma por favor que respondeste a todas as questões.
Muito obrigado pela tua imprescindível participação!
